# Drivable path detection for a mobile robot with differential drive using a deep Learning based segmentation method for indoor navigation

**DOI:** 10.7717/peerj-cs.2514

**Published:** 2024-11-19

**Authors:** Oğuz Mısır

**Affiliations:** Department of Mechatronics Engineering, Bursa Technical University, Bursa, Türkiye

**Keywords:** Mobile robots, Cybernetics system, Robotic, Navigation

## Abstract

The integration of artificial intelligence into the field of robotics enables robots to perform their tasks more meaningfully. In particular, deep-learning methods contribute significantly to robots becoming intelligent cybernetic systems. The effective use of deep-learning mobile cyber-physical systems has enabled mobile robots to become more intelligent. This effective use of deep learning can also help mobile robots determine a safe path. The drivable pathfinding problem involves a mobile robot finding the path to a target in a challenging environment with obstacles. In this paper, a semantic-segmentation-based drivable path detection method is presented for use in the indoor navigation of mobile robots. The proposed method uses a perspective transformation strategy based on transforming high-accuracy segmented images into real-world space. This transformation enables the motion space to be divided into grids, based on the image perceived in a real-world space. A grid-based RRT* navigation strategy was developed that uses images divided into grids to enable the mobile robot to avoid obstacles and meet the optimal path requirements. Smoothing was performed to improve the path planning of the grid-based RRT* and avoid unnecessary turning angles of the mobile robot. Thus, the mobile robot could reach the target in an optimum manner in the drivable area determined by segmentation. Deeplabv3+ and ResNet50 backbone architecture with superior segmentation ability are proposed for accurate determination of drivable path. Gaussian filter was used to reduce the noise caused by segmentation. In addition, multi-otsu thresholding was used to improve the masked images in multiple classes. The segmentation model and backbone architecture were compared in terms of their performance using different methods. DeepLabv3+ and ResNet50 backbone architectures outperformed the other compared methods by 0.21%–4.18% on many metrics. In addition, a mobile robot design is presented to test the proposed drivable path determination method. This design validates the proposed method by using different scenarios in an indoor environment.

## Introduction

Autonomous mobile robots (AMR) are used for tasks such as service or delivery in indoor or outdoor environments. These robots are programmed to perform their desired tasks without requiring any operator assistance. One of the main tasks of AMRs is to reach the targeted location safely and optimally. In order for mobile robots to perform these tasks, they must avoid dynamic and static obstacles in their environment (Faisal et al., 2013; Tzafestas, 2018). Lidar sensor, Visual sensor, ultrasonic proximity sensor and IR based sensors are generally used for AMRs to detect obstacles around them (Zaheer, Gulrez & Thythodath Paramabath, 2022). Using the data obtained from the sensors, methods were developed to reach the target on an optimal and safe path. Among the developed methods, the success of deep learning-based methods is also remarkable. Particularly in robotic applications, the use of deep learning has been reported to achieve successful results (Zhu & Zhang, 2021).

Deep learning methods are commonly used in AMR applications for vision-based tasks. Among these methods, the convolutional neural network (CNN) is the preferred choice for pattern classification (Badrinarayanan, Kendall & Cipolla, 2017; Sandhya Devi, Vijay Kumar & Sivakumar, 2021). Navigation applications such as object detection, semantic segmentation, instance segmentation, and classification have been developed using vision-based deep learning techniques with CNN classification (Wang, Sun & Liu, 2019).

Vision-based navigation methods aim to guide the AMR along a safe driving path by analyzing the images (Rasib et al., 2021). Semantic segmentation plays a crucial role in detecting the drivable area (Liu & Deng, 2018; Park, Yoo & Wang, 2022). This method is one of several segmentation techniques that can be used to achieve this target (Nemade & Sonavane, 2022). It is a technique used to label pixels in an image and classify objects or scenes in the image content. Semantic segmentation masks the parts of an image to be interpreted and labels the desired outputs within the images (Monasterio-Exposito, Pizarro & MacIas-Guarasa, 2022). Semantic segmentation is widely used in medical image diagnosis, robot vision, autonomous driving, and computer vision (Janai et al., 2020; Wang et al., 2020; Feng et al., 2021). These methods include fully connected network (FCN), CNN, regional CNN (R-CNN), feature pyramid network (FPN), dilated convolutions neural networks (DCNN), recurrent neural networks (RNN), attention-based models, and generative adversarial network (GAN) (Minaee et al., 2022).

The method of semantic segmentation is based on an encoder–decoder architecture (Mo et al., 2022). The encoder transforms image features into multiple levels and generates a high-dimensional feature vector. The decoder then decodes the multilevel feature vector using the high-dimensional feature vector obtained from the encoder, resulting in a labelled or segmented semantic output. Several encoder and decoder based models have been developed, including SegNet (Badrinarayanan, Kendall & Cipolla, 2017), UNet (Ronneberger, Fischer & Brox, 2015), HRNet (Yuan et al., 2019), PSPNet (Zhao et al., 2017), Deeplab (Chouai et al., 2021) family, and MaNet (Li et al., 2020). Among these, the Deeplabv3+ architecture is a powerful semantic segmentation method that uses the residual network. The methods that have been improved with Deeplabv3+ show better accuracy (Chouai et al., 2021). In a recent study, Teso-Fz-Betoño et al. (2020) applied semantic segmentation for indoor navigation of AMR using Resnet-18 transfer learning. They adopted the Deeplabv3+ encoder–decoder technique. Semantic segmentation was used to mask the path. Nguyen et al. (2022) developed an effective method for detecting drivable areas for mobile robots. They also proposed a framework that can automatically generate segmentation labels to identify road anomalies and the drivable path. In a separate study, Pan et al. (2020) presented the View Parsing Network (VPN) framework. To assess the model’s effectiveness, they applied it in a real-world setting with a mobile robot called LoCoBot.

The problem definition for the solution target of this study is as follows. Mobile robots must plan a path to reach the desired target by avoiding obstacles in their environment in the movement space. In this path-planning process, determining the drivable path that the mobile robot can follow with its wheels also means determining the robot’s movement space. After the drivable path of the mobile robot is determined, its movement space limitations are also determined.

To solve this problem, obstacle avoidance based methods using limited sensor data have been developed (Hoshino & Unuma, 2024; De Heuvel et al., 2024). However, methods that provide scene-based semantic inference using visual-based data can help mobile robots determine an efficient and safe drivable path, and semantic segmentation, which is used to extract semantic data from the scene perceived by the mobile robot, can be used effectively in navigation tasks. In fact, studies on solutions to this problem have been conducted by researchers. Explanations of this are examined in the Related Studies section. Simultaneously, semantic segmentation, which is used to extract semantic data from a scene perceived by a mobile robot, can be used effectively in navigation tasks. Researchers have addressed studies on solutions to this problem. Explanations on this are examined in the related studies section.

In this study, a semantic-segmentation-based grid-based RRT* navigation strategy method was developed to determine the drivable path of mobile robots. Gaussian Image Noise filter is used to improve segmentation estimation and multi-otsu thresholding is used to improve masked images into multiple classes. In the proposed method, the Deeplabv3+ and ResNet50 backbone model distinguishes multi-class objects and extracts drivable paths from segmented and masked images with high accuracy. The segmented image is not yet sufficient to extract the motion space of the mobile robot, and to extract the motion space of the mobile robot, the segmented images are transformed into real-world space with a perspective transform. This transformation helps to determine the path in which the mobile robot can now move with a navigation strategy. In determining the navigation strategy, the perspective transformed image is divided into grids proportional to the ground. Thus, the mobile robot could switch between these virtual grids to follow its drivable path. To ensure that the mobile robot reaches the target safely on this specified driving path, the navigation strategy was determined using the grid-based RRT* algorithm. Although the RRT* algorithm plans a path to reach the target, it requires smoothing. Smoothing was performed using the path determined by the RRT*. In addition, a mobile robot design is presented to test the proposed method. A path-following strategy is developed according to the path plan determined based on the navigation strategy developed by the mobile robot.

The contributions of this study are as follows:

• The combination of Deeplabv3+ and ResNet50 backbone multi-class semantic segmentation model is proposed for the segmentation of drivable path of mobile robots.
• A multi-class segmentation dataset consisting of indoor images was created.
• The proposed segmentation model and backbone architecture are compared with different methods in terms of performance. DeepLabv3+ and ResNet50 backbone architecture outperform the other compared methods by 0.21% to 4.18% in many metrics.
• In order to improve the segmentation success, Gaussian noise filter and multi-otsu thresholding improve the efficiency in determining the drivable path.
• After determining the drivable path by segmentation, a navigation strategy based on the grid-based RRT* algorithm is developed for the safe driving of the mobile robot to the determined final position in the real world space with perspective transform. The path determined by grid-based RRT* is smoothed to avoid unnecessary turns and deviations.
• An instructive and modular mobile robot design was developed to verify the accuracy of the proposed method and an application was developed.

The rest of this paper is organized as follows. ‘Related Works’ describes related work, ‘Overview and Problem Formulation’ describes the proposed methodology and problem formulation. ‘Experimental Evaluation and Comparisons’ presents the experiment evaluation and comparisons. Finally, ‘Conclusion’ describes the conclusion.

## Related Works

In this section, segmentation-based studies on navigation and drivable path determination applications in mobile robot applications are discussed. In particular, the focus is on the path-detection applications of deep-learning-based segmentation methods. CNN-based semantic segmentation methods are preferred for visual-based navigation studies (Matsuzaki, Masuzawa & Miura, 2022; Hyun et al., 2022).

SegNet (Badrinarayanan, Kendall & Cipolla, 2017), UNet (Ronneberger, Fischer & Brox, 2015), PSPNet (Zhao et al., 2017), MANet (Li et al., 2020) and Deeplab (Chen et al., 2016) segmentation models based on CNN-based encoder and decoder architecture can perform segmentation with high accuracy. These methods are also remarkable in their success in segmenting pixels consisting of more than one class in an image. This superior success has attracted the attention of researchers, particularly in the detection of obstacles in the navigation of mobile robots or in determining the obstacle-free path. Teso-Fz-Betoño et al. (2020) proposed an indoor navigation method with real-time semantic segmentation for indoor navigation of a mobile robot. Using the deep learning-based Resnet-18 CNN backbone network, a multi-class dataset was created with the DeepLabV3+ segmentation model to determine the obstacle avoiding path in an environment including walls, doors, and obstacles. They determined the drivable path by segmentation, considering the location of the measurement-based start and end pixel positions. In another study (Dang & Bui, 2023a), they present a real-time solution for navigation of a mobile robot in a complex challenging environment using a multi-scale fully convolutional network (FCN). They provided an effective solution by performing a perspective transformation after segmenting the image detected by the camera of the mobile robot, which defines the movement area for safe navigation. Similarly, Dang, Tran & Tan (2023) proposed a method for real-time navigation of mobile robot using a lightweight semantic segmentation model with low computational costs. They used FCN and MobilenetV2 as segmentation models. They also enhanced the input images by pre-processing the dataset with Gaussian blur and Gaussian noise. The success of the proposed method on different datasets (CitySpaces, KITTI, and Duckie) was investigated. After segmentation, a local search algorithm is designed for the mobile robot to avoid obstacles. In another study, Hyun, Woo & Kim (2022) developed a segmentation-based method for determining the drivable path of a mobile robot on the street surface in urban environments. A real-time semantic segmentation network was used with a Dynamic Context-Based Refinement Module (DCRM). Using an RGB-D camera, a dataset consisting of many classes, such as Crosswalk, Grass, and asphalt road, was used in the street environment. In another study, Nguyen et al. (2022) developed a self-supervised automatic generation segmentation label (AGSL) for determining the drivable path of an autonomous mobile robot. They used semantic segmentation to detect road anomalies and drivable roads. For this purpose, they contributed to efficient and accurate segmentation by reducing the need for manual labeling of images captured with an RGB-D camera.

Determining the drivable path of mobile robots in challenging environments is complex and requires significant processing power. The use of deep learning-based semantic segmentation to determine drivable areas facilitates the determination of movement areas. Semantic segmentation is highly effective in distinguishing elements labeled in more than one class in a given environment. The Deeplabv3+ based semantic segmentation method discussed in this study is inspired by these studies.

## Overview and Problem Formulation

**Figure 1:**
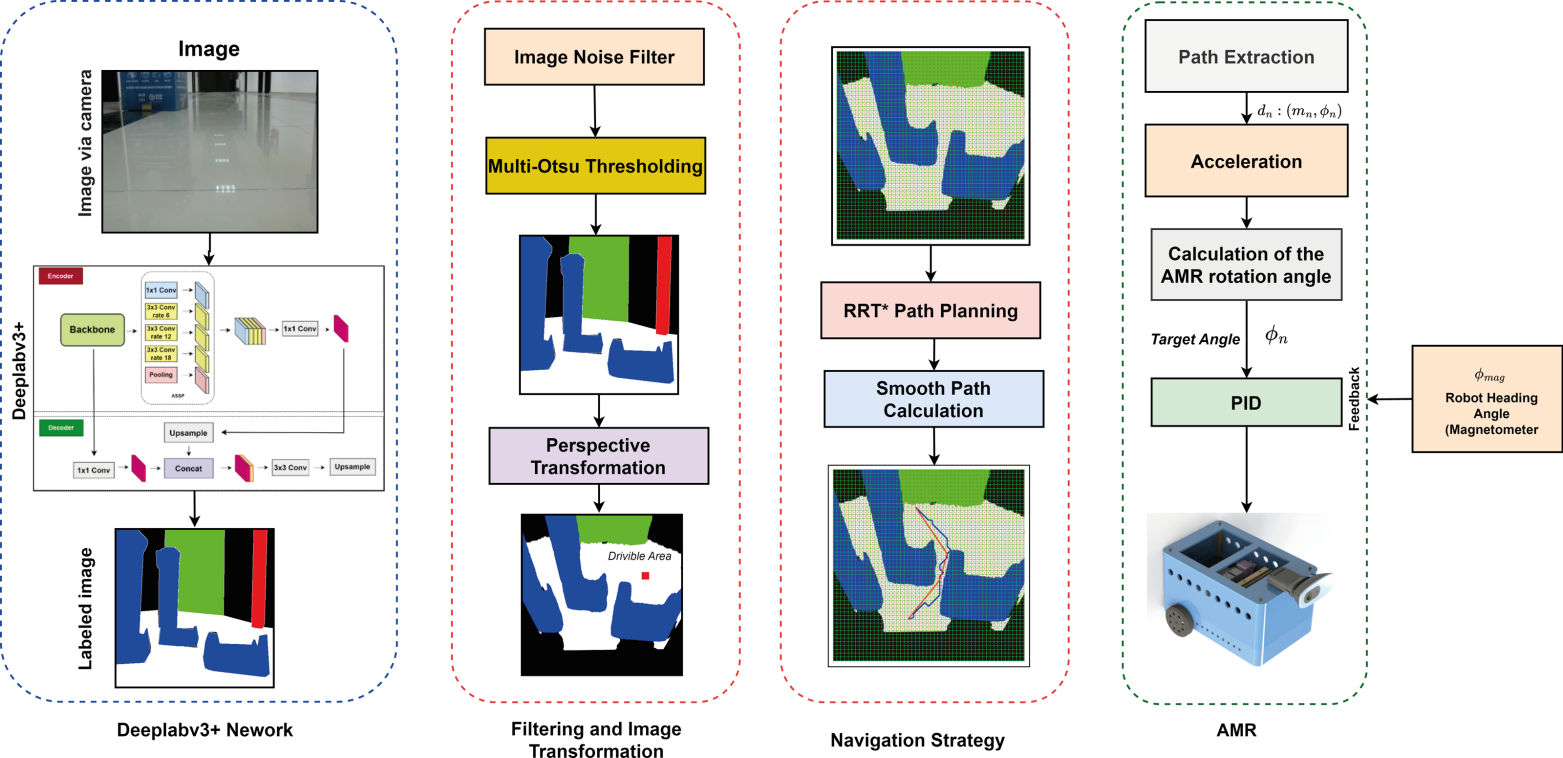
Proposed drivable path detection method.

This section presents the method developed to estimate the most suitable drivable path for AMR navigation tasks. The method extracts the drivable path by segmenting the scene image detected by the mobile robot. It also performs path planning, which enables the AMR to direct to the most suitable drivable area. Figure 1 shows a diagram of the proposed method. The first step in determining a drivable path is to mask the detected scene image using semantic segmentation. The Deeplabv3+ network was used to segment images and mask them with colors representing different classes. In the masked images, the drivable area is color white to indicate the path that the mobile robot can take. It is important to note that while the robot can move along this path, it may not always be the safest option and may not be suitable for restricted paths. To overcome these constraints, the second stage of the method involves operations that direct the mobile robot from the segmented image to a region that does not impede movement. In the second stage, the masked images are segmented with Deeplabv3+ or the noise and distortions that occur in other ways are removed. Gaussian filter was used to reduce noise in the segmented image (Mohammadi & Plataniotis, 2015). Multi-otsu thresholding was used to improve the masked images into multiple classes by segmentation after filtering the image (Gan et al., 2019). Thus, the masking complexity in multiple classes is improved. The stability of the mobile robot in terms of image input and orientation was also improved. After determining the drivable path of the mobile robot, a navigation strategy was created to direct it to its final location. Before applying the navigation strategy, a perspective transformation is performed to transform the image space of the mobile robot and the real-world space. After the perspective transformation, the image is divided into grids to plan its proportional movement with the ground. Thus, the mobile robot can switch between the grids. Then, the grid-based RRT* algorithm was applied to implement the navigation strategy to move to the final target by avoiding obstacles. Although the RRT* algorithm reaches the final target in an optimized way, the path that reaches the target is subjected to smoothing. To ensure that the mobile robot reached the existing target, AMR guidance based on PID was performed.

**Figure 2:**
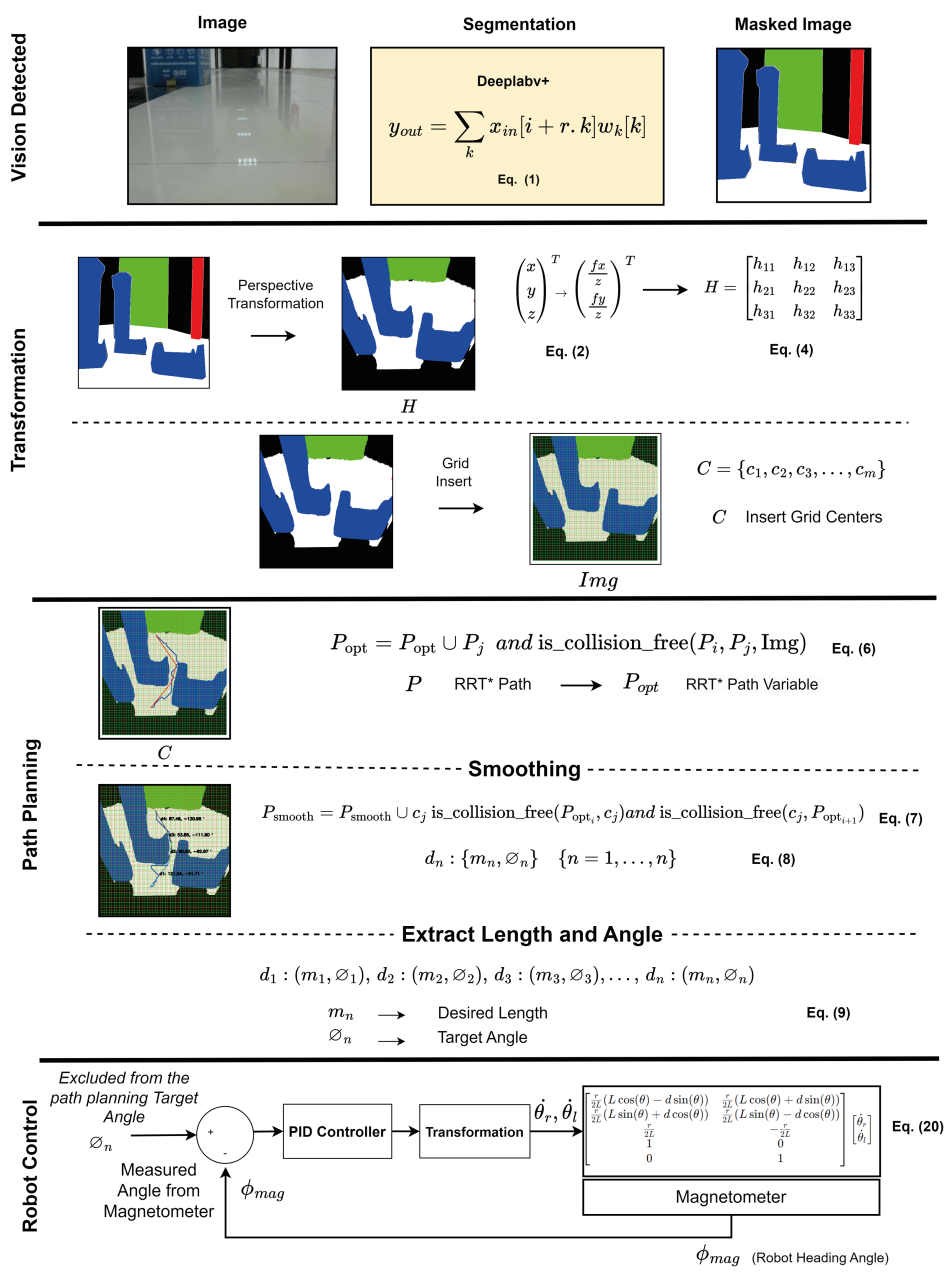
Correlation of the components used in the proposed method.

Figure 2 shows the correlation between the components used in the proposed method. The indoor navigation planning of the mobile robot using the drivable path determination approach is performed according to the vision detected, transformation, path planning and robot control stages. The details of these stages are explained in the related sections. In the vision detected stage, image detection and segmentation is performed according to Deeplabv3. Deeplabv3 segmentation is expressed in Eq. (1). In the transformation stage, pinhole transformation and four-point perspective transformation are performed as expressed in Eqs. (2) and (5) to transform between the robot’s image space and the real world space. In addition, after determining the drivable area, a grid-based navigation strategy is developed to steer the robot to the target. This navigation strategy adds grids to the masked image and the centres of these grids are determined. In the path planning phase, a path planning is extracted using the drivable area divided into grids and passing through the grid centers with RRT* as described in Eq. (6). It is then smoothed with RRT* as expressed in Eq. (7). The extracted smooth path is divided into line segments *d*_*n*_:(*m*_*n*_, ∅_*n*_) as expressed in Eq. (8). The mobile robot can be orientated to the target depending on the *m*_*n*_ lenght and ∅_*n*_ angle values extracted from these line segments as expressed in Eq. (8). In the robot control stage, the mobile robot is controlled according to the robot model expressed in Eq. (20) according to the parameters *m*_*n*_ lenght and ∅_*n*_ angle determined for the mobile robot to arrive at the target. The mobile robot rotates with the help of the PID controller to the angle reference value ∅_*n*_ determined by the path planning and the comparison of the robot head angle feedback measured with ∅_*mag*_ and moves in the direction of this angle along the value *m*_*n*_.

### Deeplabv3+ semantic segmentation network

Deeplabv3+ is a segmentation network based on encoder–decoder architecture (Chen et al., 2018). This stands out for its superior image-segmentation capability (Honarbakhsh et al., 2023). Deeplabv+3 captures multi-scale contextual information using an atrous spatial pyramid pooling (ASSP) module. It applies atrous convolution implemented for the ASSP module. The atrous convolution is expressed by Eq. (1) (Chen et al., 2018).

**Figure 3:**
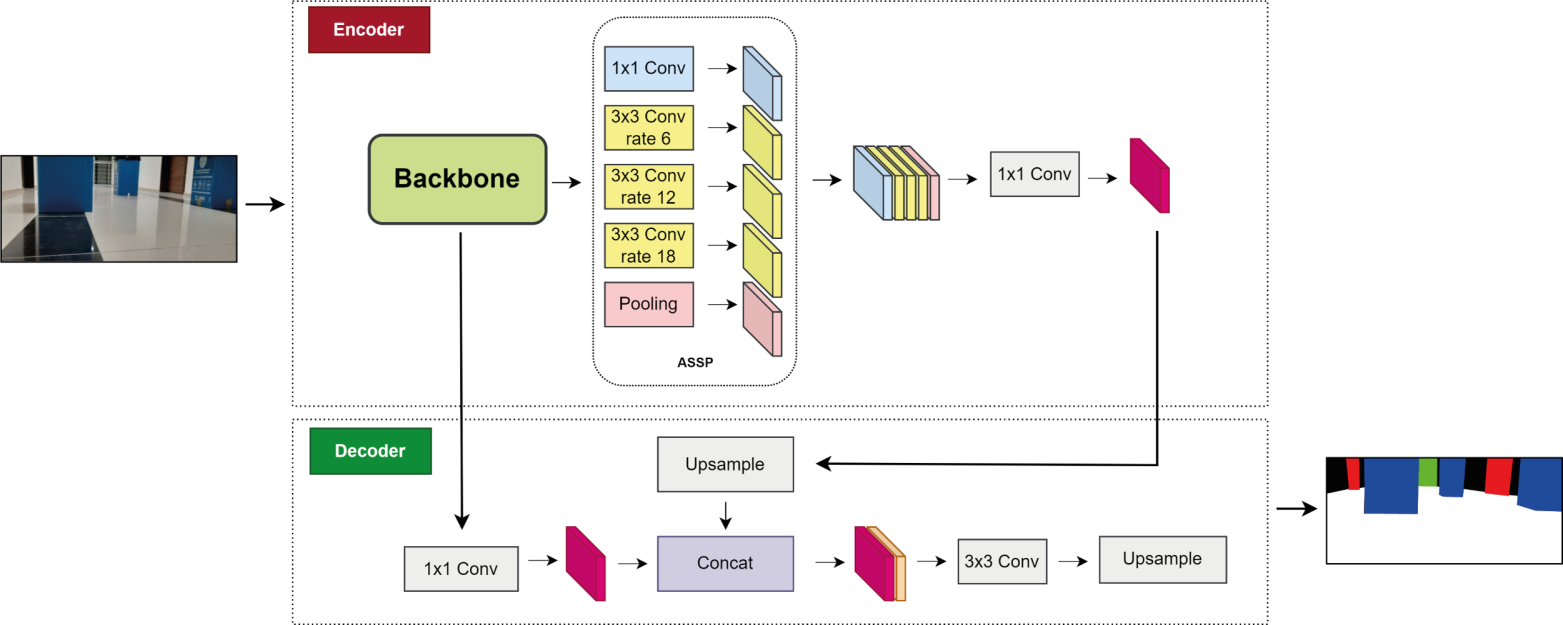
Deeplabv3+ network.

(1) \begin{eqnarray*}{y}_{out}=\sum _{k}{x}_{in} \left[ i+r.k \right] {\omega }_{k}[k]\end{eqnarray*}

where *y*_*out*_ is the output feature map at each location *i*, *x*_*in*_ is the input feature map, and *ω*_*k*_ is the convolutional filter. *r* is the stride value, also called the atrous rate. Deeplabv3+ uses both encoder and decoder architecture, and is notable for its segmentation features as well as its boundary information feature (Fu et al., 2022). Figure 3 shows the structure of Deeplabv3.

The encoder module utilizes a CNN model, such as ResNet50, ResNet11, MobileNetV2, EfficientNet, or DPN, as the backbone to extract features. The ASSP module is used to extract the features of the data and reduce the data size (Zhou et al., 2022). The ASSP module, which includes 1 × 1 convolution, 3 × 3 convolution with three rates of 6, 12, and 18, and pooling, is used to extract and reduce the data size. Depth-wise separable convolution is employed in ASSP to sample at different atrous rates and efficiently obtain multiscale information (Yang et al., 2022).

The feature maps transferred from the ASSP are merged and subjected to 1 ×1 atrous convolution, which reduces their size. The feature maps are then transferred to the decoder module, where all feature maps extracted from the backbone are also transferred and 1 × 1 convolution is applied. Feature merging is performed here, and up-sampling is applied four times to increase the size of the merged feature maps. A 3 × 3 convolution is subsequently applied for feature extraction. The sample is then sampled four times, and the final segmentation is performed. The feature map gradually returns to its initial size. Therefore, depending on the input image, the labels corresponding to the output image pixels are marked.

### Backbone architecture for feature extraction

**Figure 4:**
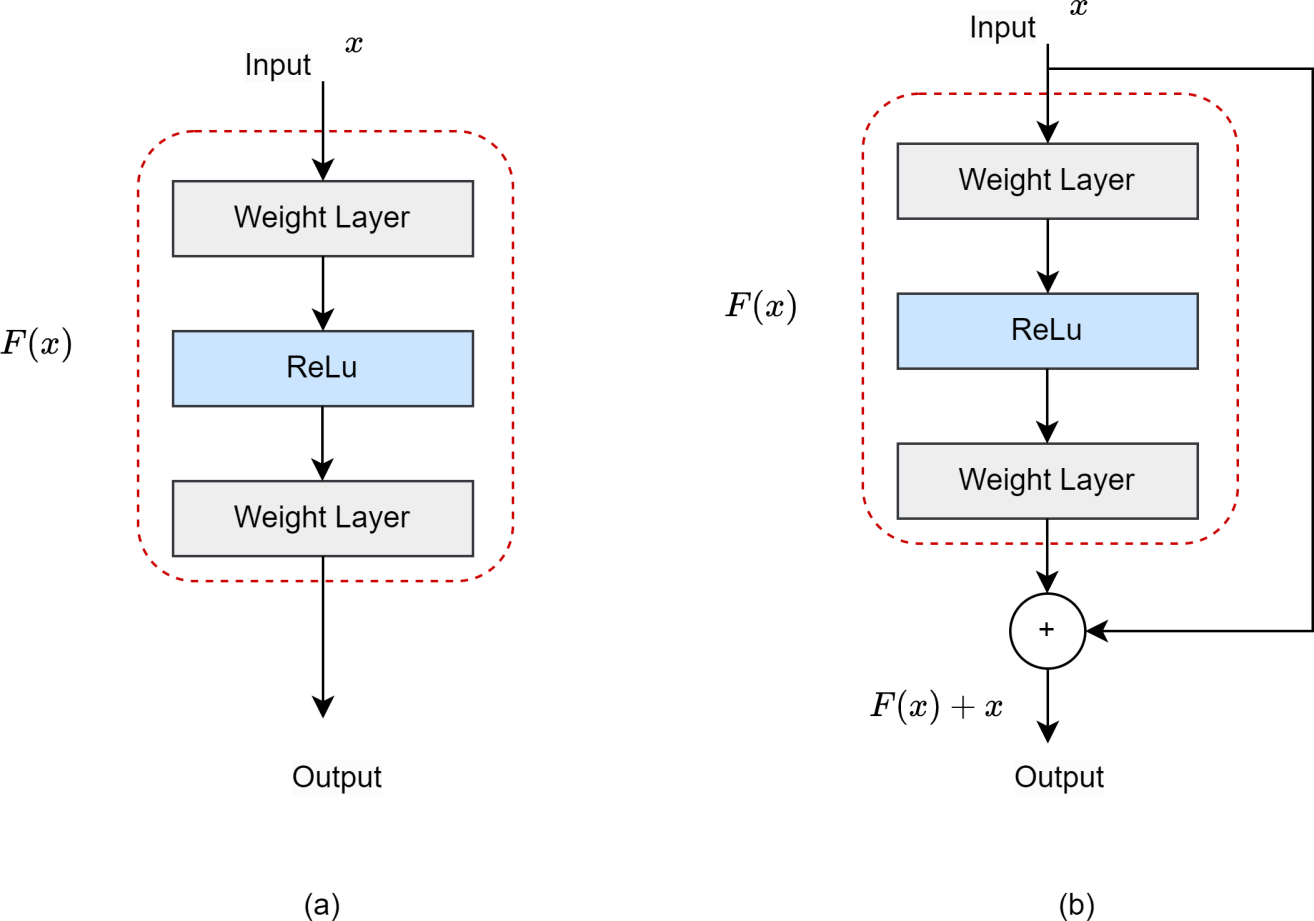
(A) Normal block. (B) Residual block.

ResNet50 is a CNN model developed specifically for object identification, and is used as the backbone network architecture (He et al., 2016). It has a 50-layer model architecture. The residual network (ResNet) was developed to prevent gradient loss and network degradation that occur in deep neural networks. Figure 4 shows the difference between a ResNet block and a normal block. The residual block includes a shortcut that jumps differently from a regular block, and jump links are added between the convolution layers to provide a connection between them.

**Figure 5:**
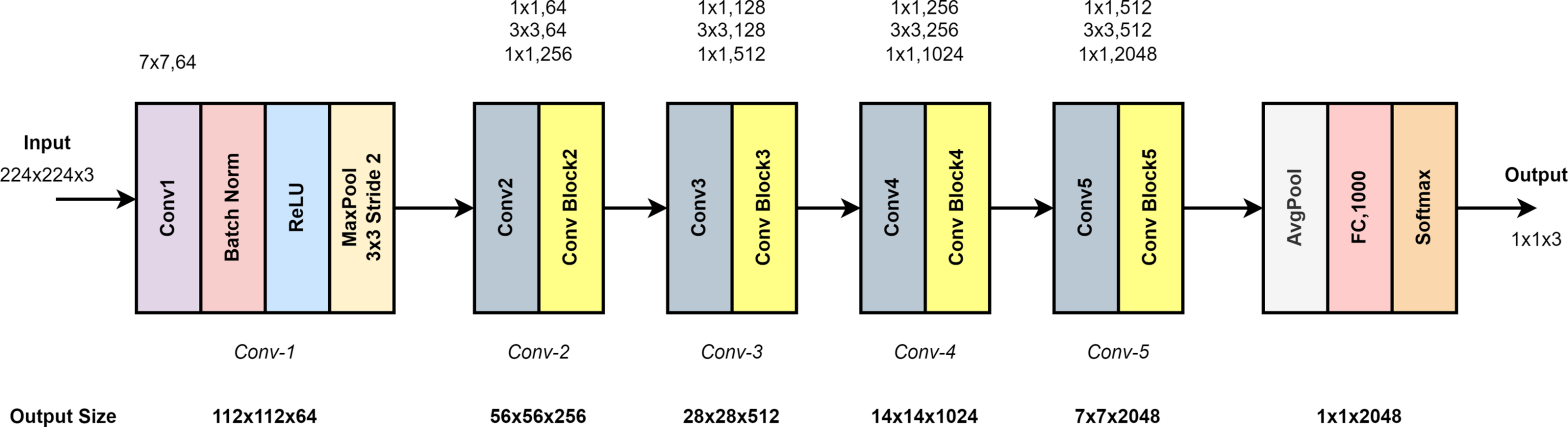
ResNet50 model architecture.

Figure 5 shows the ResNet50 model architecture. It consists of five layers: Conv1, Conv2, Conv3, Conv4 and Conv5. Conv1 in the input layer consists of 7 × 7 convolutional layers, batch normalization and 3 × 3 max pooling. As shown in Fig. 5, the architecture deepens to extract high-level features. In the last layer, average pooling and smoothing with fully connected (FC) are performed. Softmax function is used for classification (Du et al., 2023).

### Deeplabv3+ network training and data set

The Deeplabv3+ network is trained using a main backbone for drivable area segmentation of AMR. The backbone network is used for feature mapping of the drivable area. The Deeplabv3+ segmentation network is compared to UNet (Ronneberger, Fischer & Brox, 2015), feature pyramid network (FPN) (Lin et al., 2017), pyramid attention network (PAN) (Yang, Ku & Hu, 2021). Furthermore, the Deeplabv3+ network, ResNet, MobileNet, EfficientNet, and DPN backbone architectures are compared. In the comparisons, the Deeplabv3+ segmentation network and ResNet50 backbone, which have shown high performance for drivable path extraction, are used. The comparison results and backbone architecture performances are analyzed in detail in the Results section.

The Deeplabv3+ segmentation model is successful in separating differences in the scale of objects. This segmentation model is integrated with superior feature extraction capability and ResNet50 backbone network used in abstract feature extraction. In addition, Deeplabv3+ and Resnet50 combination can produce efficient segmentation results in terms of accuracy and flexibility.

The dataset used to train the Deeplabv3+ architecture was created by capturing images of different objects and scenes in a corridor (indoor). The dataset consists of 274 images. Within this dataset, 15 images are used as test data and 15 images are used as validation data. Each image is labeled using five different classes. These classes include drivable areas, walls, trash bins, doors, and windows. The classes are labeled using RGB codes. The r-g-b color codes for the classes are listed in Table 1. The images obtained from the dataset were manually colored according to the colors given in Table 1, using the auxiliary tools provided by the hatsy.ai website. In the labeling process, GT (Ground Truth) is the verified colorings that are manually labeled.

**Table 1:** Classes used for labeling.

**Labels**	**Color**	**[ r g b]**
Drivable Area	*White*	[255 255 255]
Wall	*Black*	[ 0 0 0 ]
Bin Box	*Blue*	[ 0 0 255]
Door	*Red*	[255 0 0 ]
Window	*Green*	[ 0 255 0 ]

**Figure 6:**
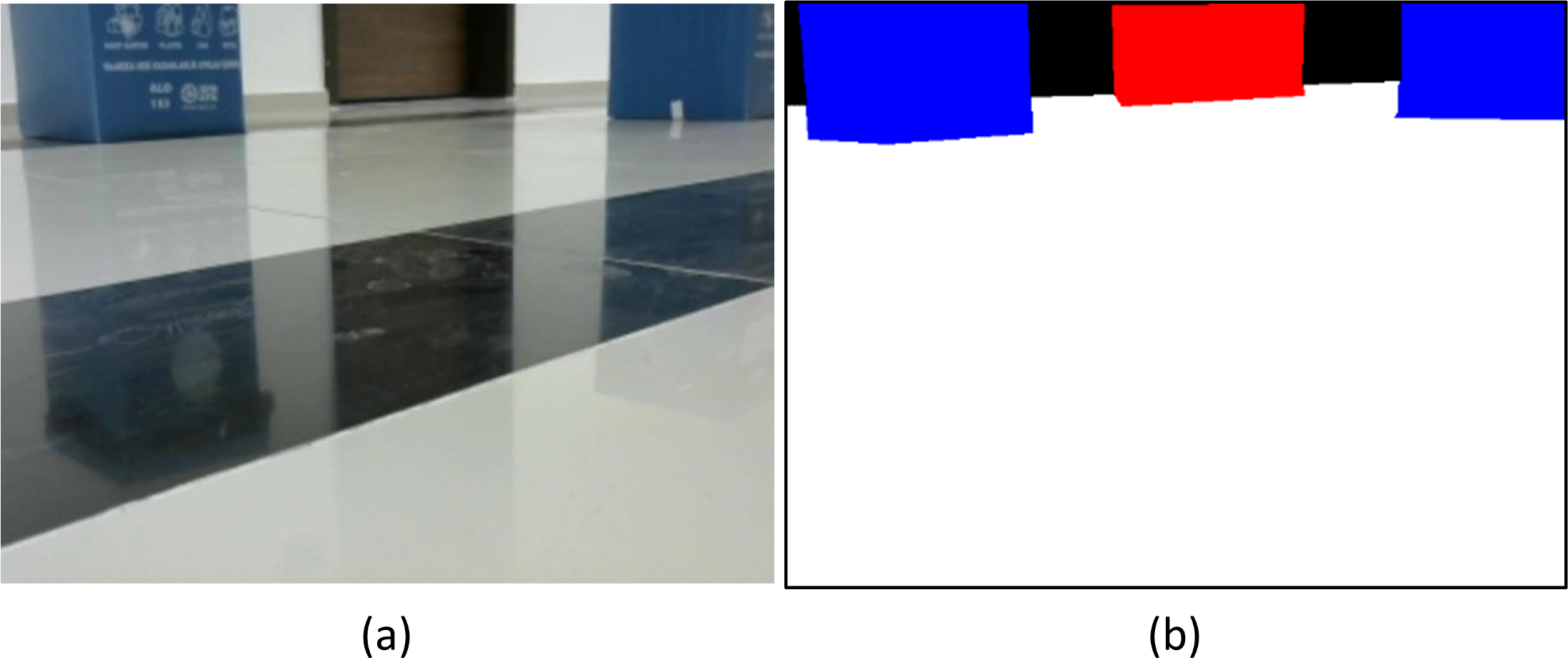
(A) GT and (B) masked image.

The dataset includes images of each class acquired at varying light levels and angles. The images are initially 640 × 480 in size but are resized to fit the input layers of the backbone networks used for training. To increase the amount of data in the dataset, augmentation techniques such as random crop, vertical flip, horizontal flip, and random rotate operations are applied to images of size 256 × 256. Figure 6 shows both normal and labeled images from the dataset. The white region in the labeled image represents the drivable path. The developed method focuses solely on detecting the drivable path, therefore images extracted from other classes are not classified as obstacles or drivable paths. To enhance their intrinsic quality, the images were labeled with multiple classes.

In this study, the Deeplabv3+ network and other compared networks were trained based on different backbone architectures. Labeled and normal (GT) images were used for training. The hyperparameters used for training are listed in Table 2. The segmentation methods and the backbone networks are trained with the same hyperparameters given in Table 2. Thus, the performance of the segmentation methods and backbone networks on the same hyperparameters is analyzed.

**Table 2:** Hyperparameters.

**Training parameters**	**Values**
Segmentation Network	Deeplabv3+,Unet,FPN,PAN,PSPNet
Backbone	ResNet, MobileNet, EfficientNet, DPN, FPN
Activation	Softmax2d
Epoch	200
Optimization	Adams
Learning Rate	0.0001
Loses Function	Dice Loss
Shuffle	True
Batch Size	10

### Navigation strategy and drivable path detection

In the proposed method, the drivable area of the mobile robot is extracted from images segmented with Deeplabv3+. Since the Deeplabv3+ network can perform segmentation with high accuracy, the drivable area that allows the mobile robot to avoid obstacles is also extracted. Compared to other models, the Deeplabv3+ segmentation model learns details in images by covering a wider area. This allows the model to better understand the objects and edges. This is one of the reasons for selecting this model. In a complex environment with obstacles, obstacle detection is not as easy as it seems and requires a complex process. The drivable area in the segmented images was masked in white. A navigation strategy is required to ensure that the mobile robot avoids obstacles in the drivable area. The navigation strategy in the proposed approach consists of the following steps. (1) After segmentation to detect drivable areas, perspective transformation is performed to perform the transformation and adjustment process between the image space of the mobile robot and the real world. (2) By adjusting the grid-based proportional precise navigation coordination after the perspective transformation, inspired by the work of Dang & Bui (2023b) (3) After grid-based surface proportional navigation coordination, path planning is performed with grid-based RRT*, and the path is smoothed. RRT* determines the path by switching between the centers of the proportional grids using the path-planning algorithm. In short, the mobile robot moved by switching between the centers of the grid. The transition of the mobile robot between the centers of the grids was experimentally determined.

### Perspective transformation and grid-based navigation coordination

**Figure 7:**
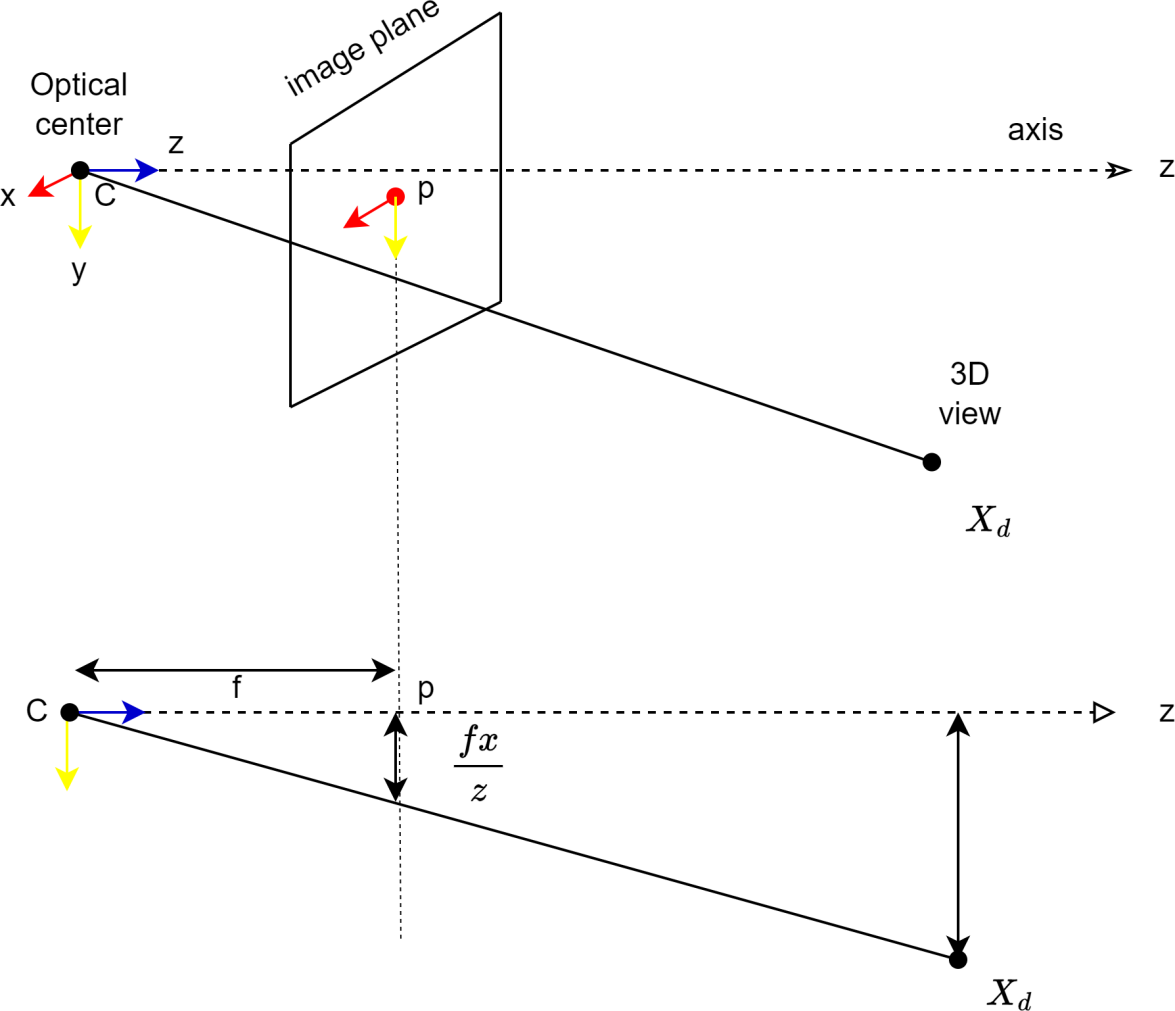
Coordination of the camera with the image surface.

The pinhole model is used to coordinate the image of the camera placed in front of the mobile robot with the ground surface. The pinhole model also mathematically explains how a point in the 3D world will be adapted to the 2D image plane. Figure 7 shows the pinhole model. Here, the plane *z* = *f* is referred to as the image plane or focal plane. According to the pinhole model, a point at coordinate ${ \left( x,y,z \right) }^{T}$ is mapped to point ${ \left( \frac{fx}{z} , \frac{fy}{z} ,f \right) }^{T}$ in the image plane as shown in Fig. 7. The transformation from three-dimensional space to two-dimensional space is done as expressed in Eq. (2) (Hartley & Zisserman, 2004).

Here *C* is the optical centre of the camera, *p* is the image plane and *X*_*d*_ is the point in 3D space. *x* is the point where the line to the projection centre coincides with the image plane.

(2) \begin{eqnarray*}{ \left( x,y,z \right) }^{T}\rightarrow { \left( \frac{fx}{z} , \frac{fy}{z} \right) }^{T}.\end{eqnarray*}

The linear transition between the center projection coordinates of the points of the surface viewed by the camera can be expressed by matrix multiplication. The transformation from the world coordinate system to the image coordinate according to the pinhole transformation is expressed in Eq. (2) (Hartley & Zisserman, 2004). *K* here refers to the camera parameters.

(3) \begin{eqnarray*}x=K \left[ Rt \right] {X}_{d}.\end{eqnarray*}

Here *I* is the internal orientation of the camera, R and t are matrices expressing the orientation of the camera and its position with respect to the world coordinate system.

After transferring the real world plane to the image plane, perspective transformation can be used to obtain the top view image. With perspective transformation, the mobile robot can plan the path to avoid obstacles in the real world plane.

When you want to make perspective correction on an image, the image plane (*x*, *y*) and the real world plane $ \left( {x}^{{}^{{^{\prime}}}},{y}^{{}^{{^{\prime}}}} \right) $ are transformed. The perspective transformation relationship function can be calculated using the four corner points (Dang & Bui, 2023a). For this, homography is used to perform perspective correction on the image. The homography matrix [*H*] is expressed in Eq. (5) (Lamovsky, 2013).

(4) \begin{eqnarray*}H= \left[ \begin{array}{@{}ccc@{}} \displaystyle {h}_{11}&\displaystyle {h}_{12}&\displaystyle {h}_{13}\\ \displaystyle {h}_{21}&\displaystyle {h}_{22}&\displaystyle {h}_{23}\\ \displaystyle {h}_{31}&\displaystyle {h}_{32}&\displaystyle {h}_{33} \end{array} \right] .\end{eqnarray*}

In the perspective transformation, the homography matrix is used and $ \left( {x}^{{}^{{^{\prime}}}},{y}^{{}^{{^{\prime}}}} \right) $ transformation is performed as in Eq. (5).

(5) \begin{eqnarray*}\begin{array}{@{}c@{}} \displaystyle {x}^{{^{\prime}}}= \frac{{h}_{11}x+{h}_{12}y+{h}_{13}}{{h}_{31}x+{h}_{32}y+{h}_{33}} \end{array}~~~~~~~~{y}^{{^{\prime}}}= \frac{{h}_{21}x+{h}_{22}y+{h}_{23}}{{h}_{31}x+{h}_{32}y+{h}_{33}} .\end{eqnarray*}

**Figure 8:**
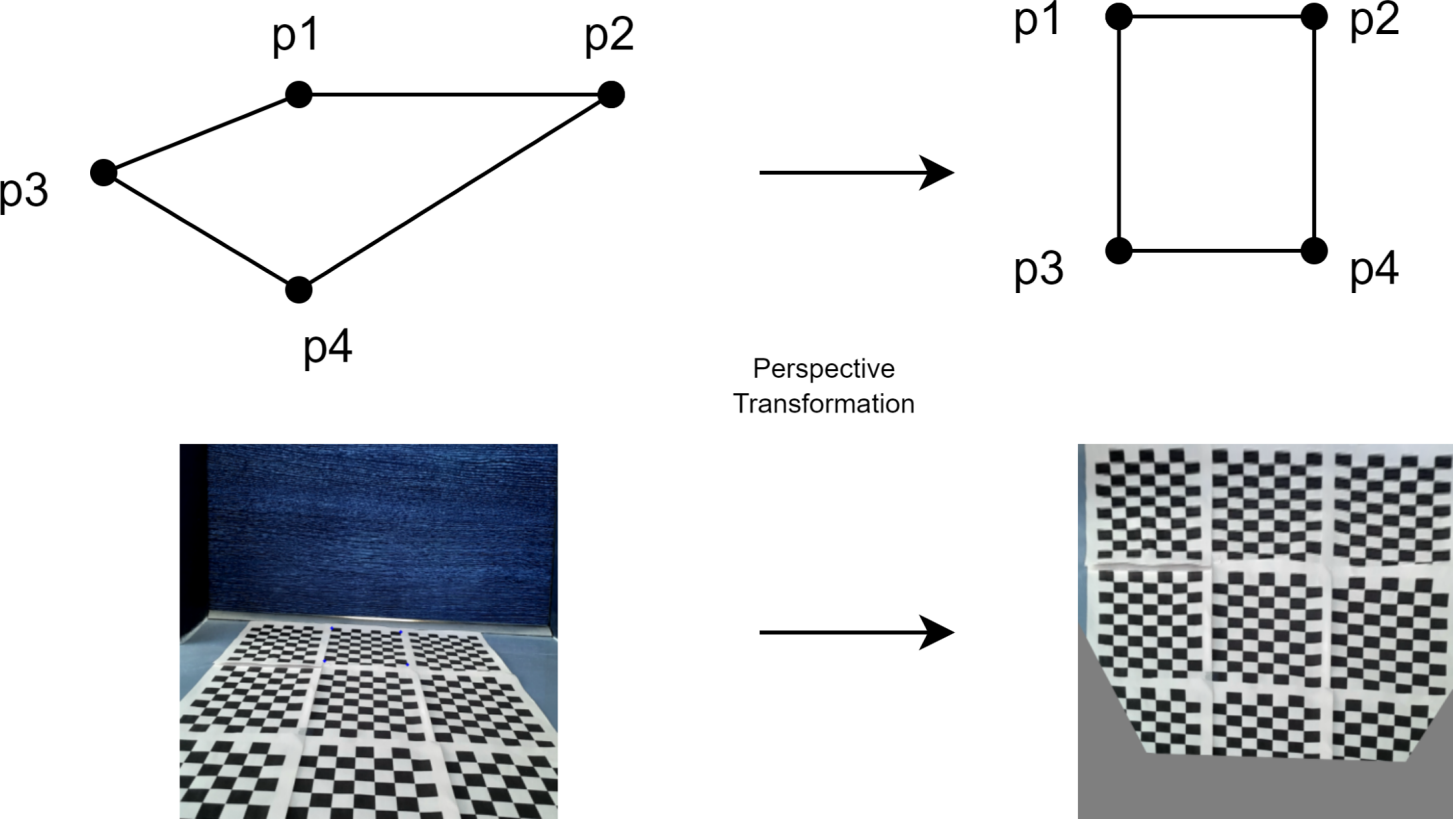
Perspective transformation according to four points.

Figure 8 shows the transformation of the front view of the mobile robot to the top view to express the perspective transformation. In the transformation, the checkerboard image is taken from the front view of the mobile robot and the perspective transformation is made according to four points for the top view of the checkerboard.

### Path planning

**Figure 9:**
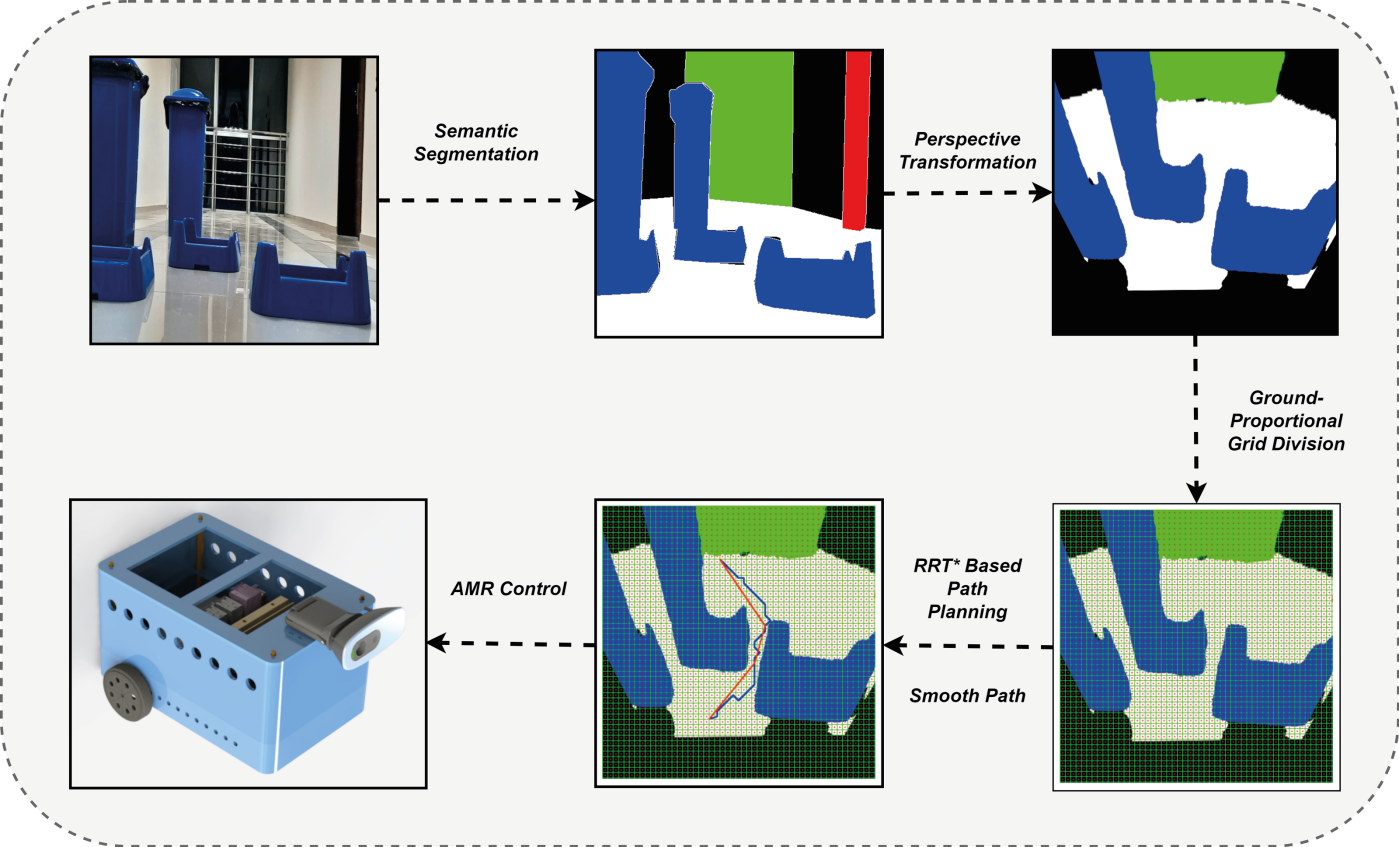
Semantic segmentation and path planning process.

After perspective correction, grid division is performed proportionally to the ground so that path planning can be done with the top view. Thus, the mobile robot can proportionally move between the experimentally determined grids. Path planning can now be done in a way that the mobile robot can see the ground from above. The grid-based RRT* algorithm is used to go to the targeted location based on the perspective transformed and ground proportional grid-divided image (Chao et al., 2018). Figure 9 shows the path planning process according to the segmentation process.

According to the grid-based RRT* algorithm, path planning is performed according to the perspective view. Thus, the path is determined for the mobile robot to reach the targeted location by avoiding obstacles. RRT uses a strategy to pass through the center points of adjacent grids while planning the path. Although the grid-based RRT* algorithm is optimized to reach the target, the path needs to be smooth due to the transitions between grids. For this, the smooth process is performed. The smooth process is performed in two steps. These are optimizing and insert grid center. The extraction of the optimized path is expressed in Eq. (6), where *P* = *P*_0_, *P*_1_, …, *P*_*n*_ is the path extracted by grid-based RRT*, *P*_*opt*_ is the variable used to extract the optimized path and *Img* is the image used for collision control including the grid centers. The used *is*_*collision*_*free*(*P*_*i*_, *P*_*j*_, *Img*) function checks whether the optimized path extracted from the perspective transformed image has a collision with obstacles. If there is no collision between the points *P*_*i*_, *P*_*j*_ in the collision control, this point is added to the optimized path.

(6) \begin{eqnarray*}\begin{array}{@{}ll@{}} \displaystyle {P}_{opt}={P}_{opt}\mathrm{U}{P}_{j}&\displaystyle is\text{_}collision\text{_}free({P}_{i},{P}_{j},Img) \end{array}.\end{eqnarray*}

After the smooth process, the second stage is applied to add the optimized path to the grid centers. In this stage, each center point is checked and the path is transformed into a smoother form. As expressed in Eq. (7), the insert grid center is made. Here *C* = *c*_1_, *c*_2_, *c*_3_, …..*c*_*m*_ refers to the grid centers. *P*_*smooth*_ inserts grid center refers to the path. Where *m* is the number of centres. *P*_*smooth*_ inserts grid center refers to the path. *P*_*smooth*_ consists of line segments, where *P*_*smooth*_ = *d*_0_, *d*_1_, …, *d*_*n*_, where n is the line segment.

(7) \begin{eqnarray*}\begin{array}{@{}ll@{}} \displaystyle {P}_{smooth}={P}_{smooth}\mathrm{U}{c}_{j}&\displaystyle is\text{_}collision\text{_}free({{P}_{opt}}_{i},{c}_{j})\text{and}is\text{_}collision\text{_}free \left( {c}_{j},{{P}_{opt}}_{i+1} \right) \end{array}.\end{eqnarray*}

It is checked whether there is a collision between the grid centers and the existing path segments. If there is no collision, the grid center is added as a new path. Motion planning is performed based on the acceleration sensor and magnetometer to determine the movement of the mobile robot according to the determined path planning. According to the angle and distance information obtained from the path planning, the mobile robot can plan its movement using the acceleration and magnetometer. For this purpose, the angle and distance information of the straight lines connected to the grid centers and extending to the final target are extracted on the smooth path.

Since *P*_*smooth*_ can consist of many line segments, it is expressed as *d*_*n*_:*m*_*n*_, ∅_*n*_ in Eq. (8). *P*_*smooth*_ as expressed in Eq. (7), where *P*_*opt*_ is the optimized path and C is the smooth path determined with respect to the grid centers. In Eq. (8) the line segments *d*_*n*_ forming *P*_*smooth*_ are expressed.

(8) \begin{eqnarray*}{d}_{n}:{m}_{n},{\varnothing }_{n}n=1\ldots n.\end{eqnarray*}

The straight line segments extracted on the path determined as smooth are expressed as in Eq. (9). These line segments are determined by the number of line segments n extracted as expressed in Eq. (9). Here *m*_*n*_ is the length of the line segment and ∅_*n*_ is its angle.

(9) \begin{eqnarray*}{d}_{1}:({m}_{1},{\varnothing }_{1}),{d}_{2}:({m}_{2},{\varnothing }_{2}),{d}_{3}:({m}_{3},{\varnothing }_{3}),\ldots ,{d}_{n}:({m}_{n},{\varnothing }_{n}).\end{eqnarray*}

At the same time, *m*_*n*_ is the length of the path that the mobile robot has to take and ∅_*n*_ is the orientation angle extracted from the path planning.

**Figure 10:**
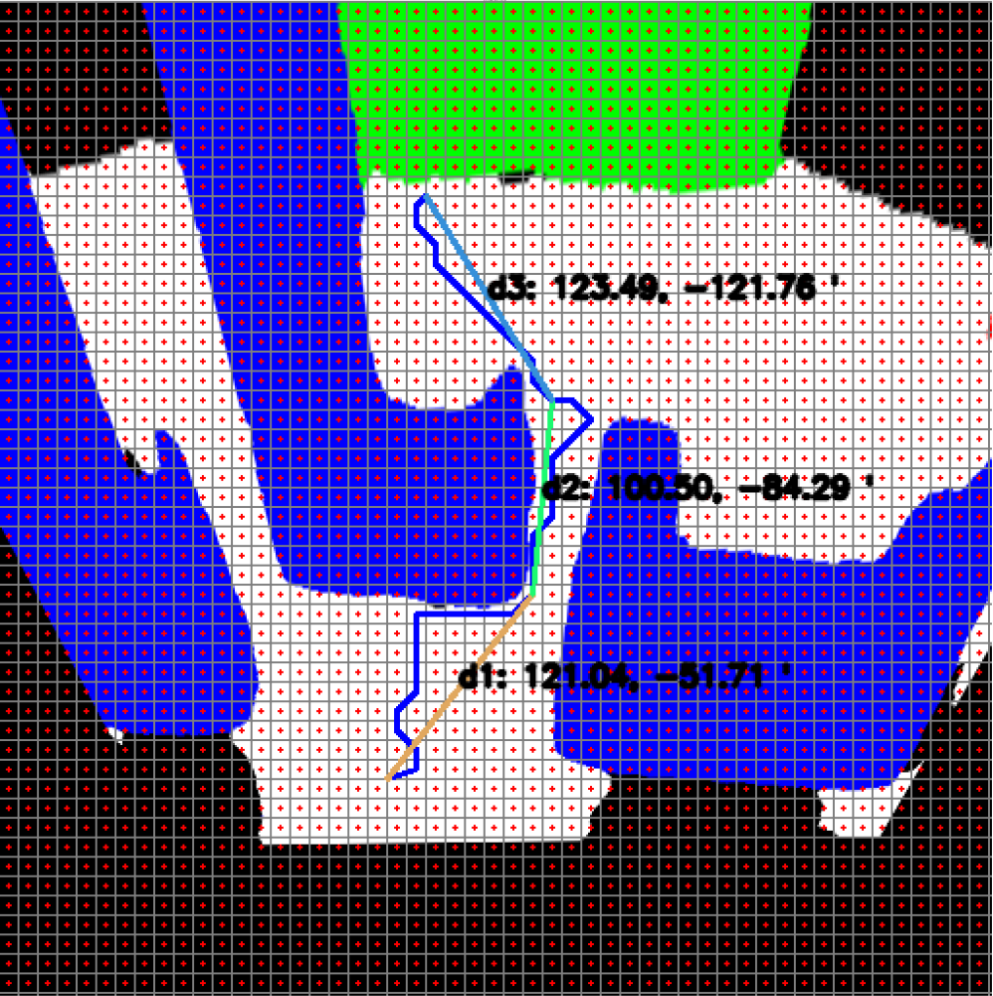
Angle and distance information of the smooth path.

The line segments expressed here have angle and distance information. Distance and angle information is expressed as *d*_1_:(*m*_1_, ∅_1_). Figure 10 shows the angle and distance information of an example smooth path for which path planning is given. A magnetometer is used to ensure that the mobile robot turns to the correct angle of its part.

A magnetometer and acceleration sensor are used to enable the mobile robot to plan its motion on a smooth path. The acceleration sensor is used to ensure that the mobile robot moves on a straight line. To control the rotation of AMR to the targeted angel ∅_*n*_, the current angle information of the mobile robot is measured by a magnetometer. To control the rotation of the AMR to the targeted angel ∅_*n*_, the current angle information of the mobile robot is measured with a magnetometer. AMR is directed to the targeted angle with the help of a PID controller.

### Mobile robot design, mathematical model and sensors

**Figure 11:**
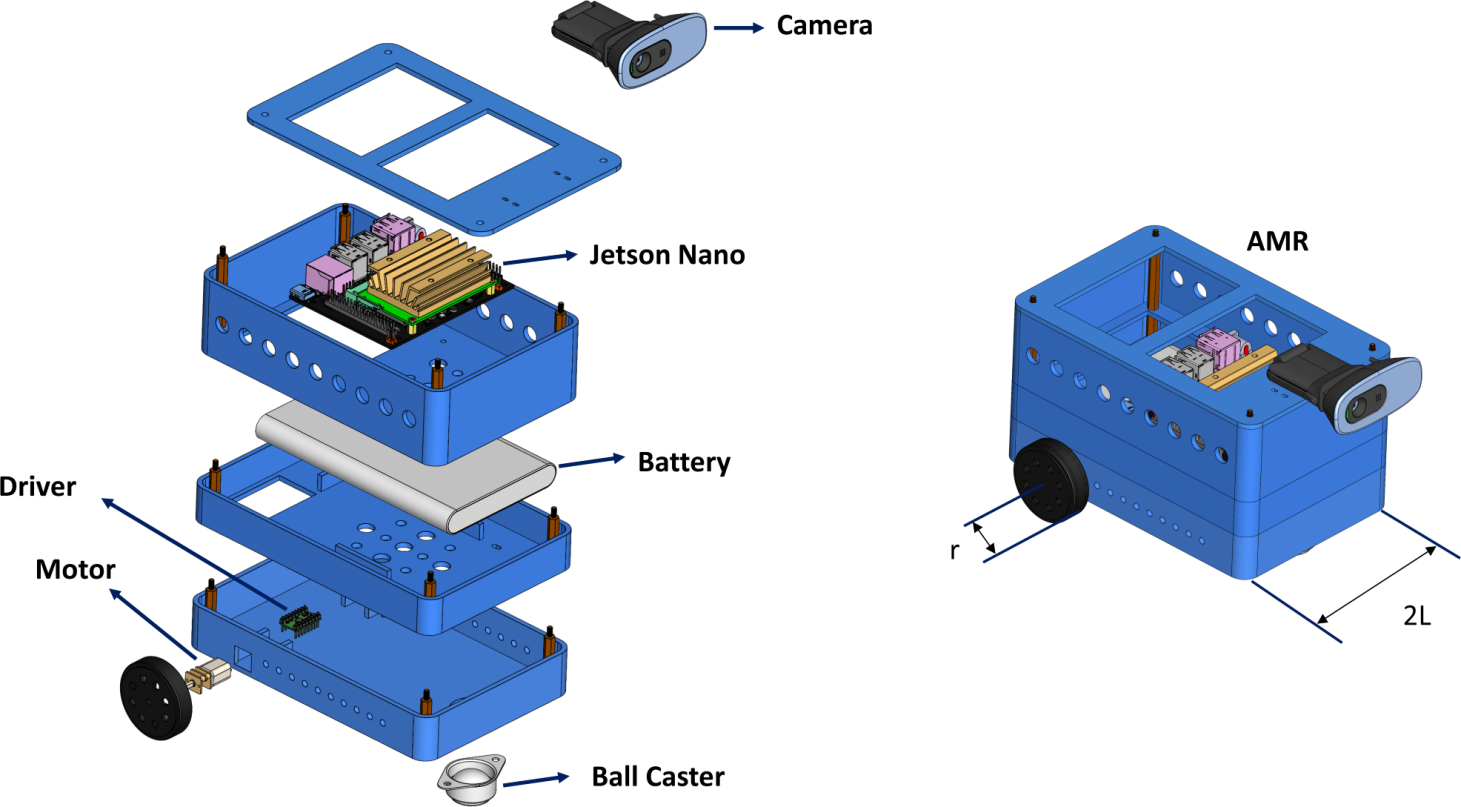
Instructive and modular AMR architecture.

In this study, a two-wheel AMR is developed to implement the proposed method. The developed AMR is shown in Fig. 11. The AMR is driven by a two-wheel differential. The distance between the two wheels is 2*L* = 121 *mm*. The AMR is equipped with a monocular camera. The monocular camera captures images with a resolution of 480  ×  640.

**Figure 12:**
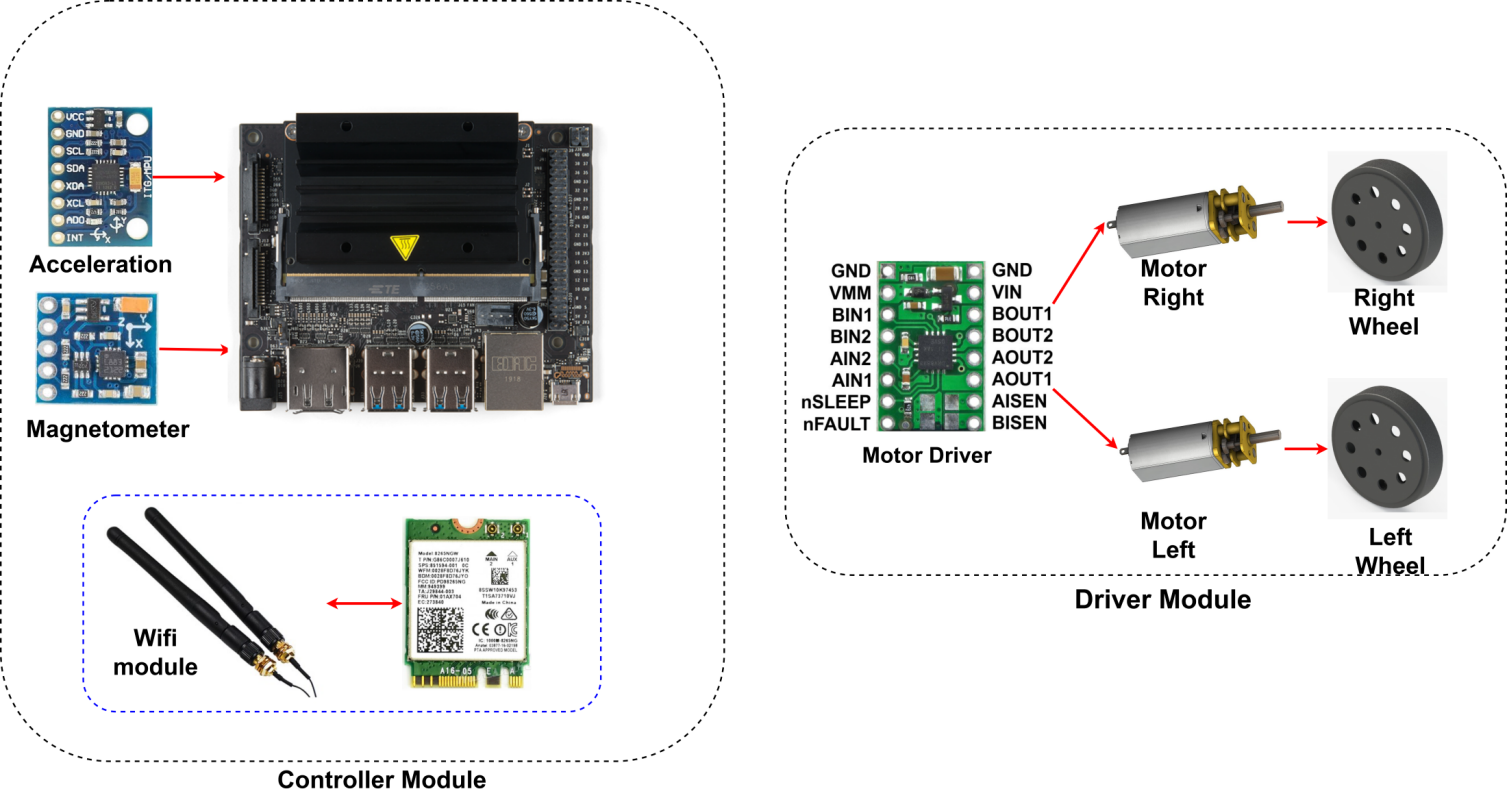
AMR hardware.

Nvidia’s Jetson Nano is used as the control unit. The Jetson Nano, a Deeplabv3+ based drivable path segmentation method, acts according to the inputs it receives from cameras and sensors. The Nvidia Jetson Nano is a development platform for artificial intelligence applications. It is preferred for real-time applications. It shows high performance in deep learning applications. The Jetson Nano has 128 CUDA cores. It also has a 4-core 1.43 GHz ARM A57 processor. The Nvidia Jetpack 4.5 SDK was preferred for the development of the Jetson Nano used in this study. Figure 12 shows the control and driver modules of AMR. In the control module, AMR implements the proposed method using Jetson Nano. The Jetson Nano module is powered by a 5V, 2A 10000 mAh portable battery. The current heading angle, which is input as feedback to the PID controller specified in the proposed method, is detected using a magnetometer. The magnetometer used is the HMC58832L. It communicates with the Jetson Nano using I2C communication protocol. Low noise 12-bit ADC +/- 1.3 to 8 Gauss field resistance measurement range. It provides a fast 160 Hz output rate. MPU6050 inertial measurement unit (IMU) is used as the acceleration sensor. It has a three-axis accelerometer and three-axis gyroscope features.

 A Wi-Fi module is used for remote programming and data monitoring. The WLAN module model is AC8265. It connects directly to the Jetson nano. It has 2.4Ghz and 5Ghz dual band capability. However, 2.4Ghz was used in this study. It can communicate at a maximum speed of 867 Mbps. The driver module consists of a motor driver unit for controlling the left and right wheels of the AMR. The driver card (DRV8835) used for motor control moves the left and right wheels back and forth depending on the PWM duty cycle ratio. The DRV8835 can drive dual H-bridge motors. It provides 1.2A continuous and 1.5A continuous current support per channel. The operating voltage is 0–11. Low voltage, high current and high temperature protection are available. Two pieces 6V, 350 rpm 15 mm reducer DC motors are connected to the driver.

**Figure 13:**
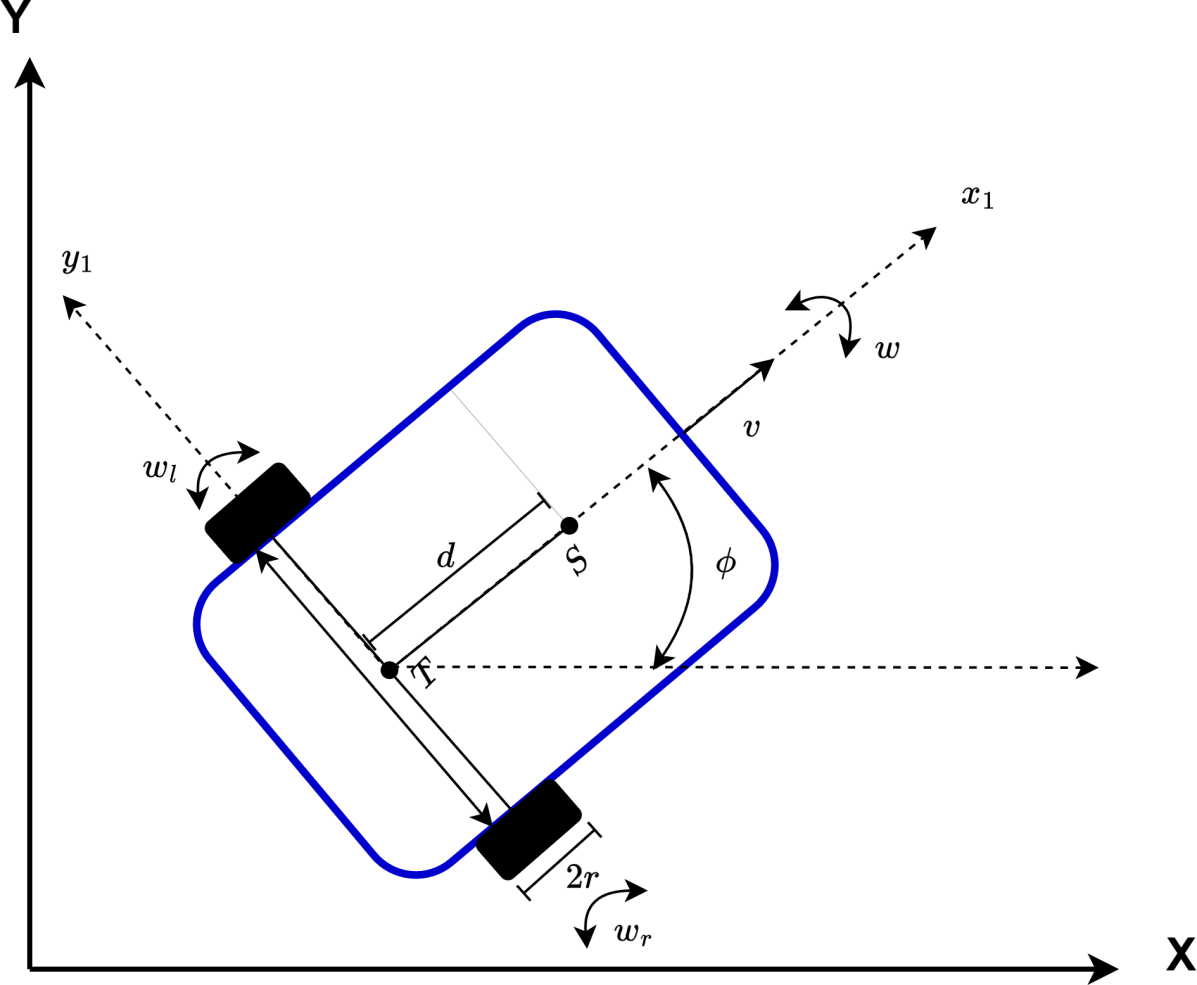
Representation of the configuration of AMR.

The AMR has a two-wheeled non-holonomic model. The configuration of the AMR is shown in Fig. 13. The center of gravity of the AMR is expressed by *S*. The intersection point of the axis passing through the centers of the wheels and the center of the axis passing through the center of the AMR is expressed by *T*. The distance between *T* and *S* is expressed as *d*. The angular velocity is w and the linear velocity is *v*. In addition, the angular velocities of the left and right wheels of the AMR are expressed as *w*_*l*_ and *w*_*r*_, respectively.

Equation (10) specifies the position and angle of the non-holonomic mobile robot in the 2D plane. Here *q* denotes the *x* and *y* position of the AMR and ∅ denotes the heading angle.

(10) \begin{eqnarray*}q= \left( \begin{array}{@{}c@{}} \displaystyle x\\ \displaystyle y\\ \displaystyle \varnothing \\ \displaystyle {\theta }_{r}\\ \displaystyle {\theta }_{l} \end{array} \right) .\end{eqnarray*}

Equation (10) specifies the position and head angle of the AMR depending on its velocity and angular velocity. *v* is the velocity and *w* is the angular velocity of the mobile robot. Equation (11) is used to calculate the speed of the AMR (Ali et al., 2021). Here *r* is the wheel radius.

(11) \begin{eqnarray*}v= \frac{r. \left( {\dot {\theta }}_{r}+{\dot {\theta }}_{l} \right) }{2} = \frac{r. \left( {w}_{r}+{w}_{l} \right) }{2} .\end{eqnarray*}

In addition, the velocities of each wheel can be expressed as in Eqs. (12)–(14) and the robot’s velocity *v* can be calculated based on these velocities (Mondal et al., 2022).

(12) \begin{eqnarray*}{v}_{r}& =r.{w}_{r}\end{eqnarray*}

(13) \begin{eqnarray*}{v}_{l}& =r.{w}_{l}\end{eqnarray*}

(14) \begin{eqnarray*}v& = \frac{{v}_{r}+{v}_{l}}{2} .\end{eqnarray*}

The angular velocity of the AMR and the angular velocities of the right and left wheels are expressed in Eqs. (15)–(17).

(15) \begin{eqnarray*}w& =\dot {\varnothing }= \frac{r \left( {\dot {\theta }}_{r}+{\dot {\theta }}_{l} \right) }{2L} \end{eqnarray*}

(16) \begin{eqnarray*}{\dot {\theta }}_{r}& = \frac{2v}{r} + \frac{w}{r} \end{eqnarray*}

(17) \begin{eqnarray*}{\dot {\theta }}_{l}& = \frac{2v}{r} - \frac{w}{r} .\end{eqnarray*}

In the AMR kinematic model, the drifts caused by the wheels are ignored. Due to non-holonomic constraints, the linear velocity of the robot in the *y*-axis is assumed to be zero since it moves in a 2D plane. This restricts the sideways movement of the robot. Depending on this situation, the constraint equations of the robot are expressed in Eqs. (18)–(20).

(18) \begin{eqnarray*}\dot {y}\cos \nolimits \left( \varnothing \right) -\dot {x}sin \left( \varnothing \right) -\dot {\varnothing }d& =0\end{eqnarray*}

(19) \begin{eqnarray*}\dot {x}\cos \nolimits \left( \varnothing \right) +\dot {y}\sin \nolimits \left( \varnothing \right) +L.\dot {\varnothing }-r{\dot {\theta }}_{r}& =0\end{eqnarray*}

(20) \begin{eqnarray*}\dot {x}\cos \nolimits \left( \varnothing \right) +\dot {y}\sin \nolimits \left( \varnothing \right) -L.\dot {\varnothing }-r{\dot {\theta }}_{l}& =0\end{eqnarray*}

Equation (21) is used to calculate the position, heading angle, right and left wheel angular velocities based on the non-holonomic constraints of the mobile robot (Nandy et al., 2011). In the applications, *v* is constant and 20 mm/sec. AMR can determine the final position based on ${\dot {\theta }}_{r}$ and ${\dot {\theta }}_{l}$.

(21) \begin{eqnarray*} \left[ \begin{array}{@{}c@{}} \displaystyle \dot {x}\\ \displaystyle \dot {y}\\ \displaystyle \begin{array}{@{}c@{}} \displaystyle \varnothing \\ \displaystyle \begin{array}{@{}c@{}} \displaystyle {\theta }_{r}\\ \displaystyle {\dot {\theta }}_{l} \end{array} \end{array} \end{array} \right] = \left[ \begin{array}{@{}cc@{}} \displaystyle \frac{\mathrm{r}}{2L} (\mathrm{L}.\cos \nolimits \left( \varnothing \right) -dsin \left( \varnothing \right) )&\displaystyle \frac{\mathrm{r}}{2L} (\mathrm{L}.\cos \nolimits \left( \varnothing \right) +dsin \left( \varnothing \right) )\\ \displaystyle \\ \displaystyle \frac{\mathrm{r}}{2L} (\mathrm{L}.\sin \nolimits \left( \varnothing \right) +dcos \left( \varnothing \right) )&\displaystyle \frac{\mathrm{r}}{2L} (\mathrm{L}.\sin \nolimits \left( \varnothing \right) -dcos \left( \varnothing \right) )\\ \displaystyle \frac{r}{2L} &\displaystyle - \frac{r}{2L} \\ \displaystyle 1&\displaystyle 0\\ \displaystyle 0&\displaystyle 1 \end{array} \right] \left[ \begin{array}{@{}c@{}} \displaystyle {\dot {\theta }}_{r}\\ \displaystyle {\dot {\theta }}_{l} \end{array} \right] .\end{eqnarray*}

**Figure 14:**
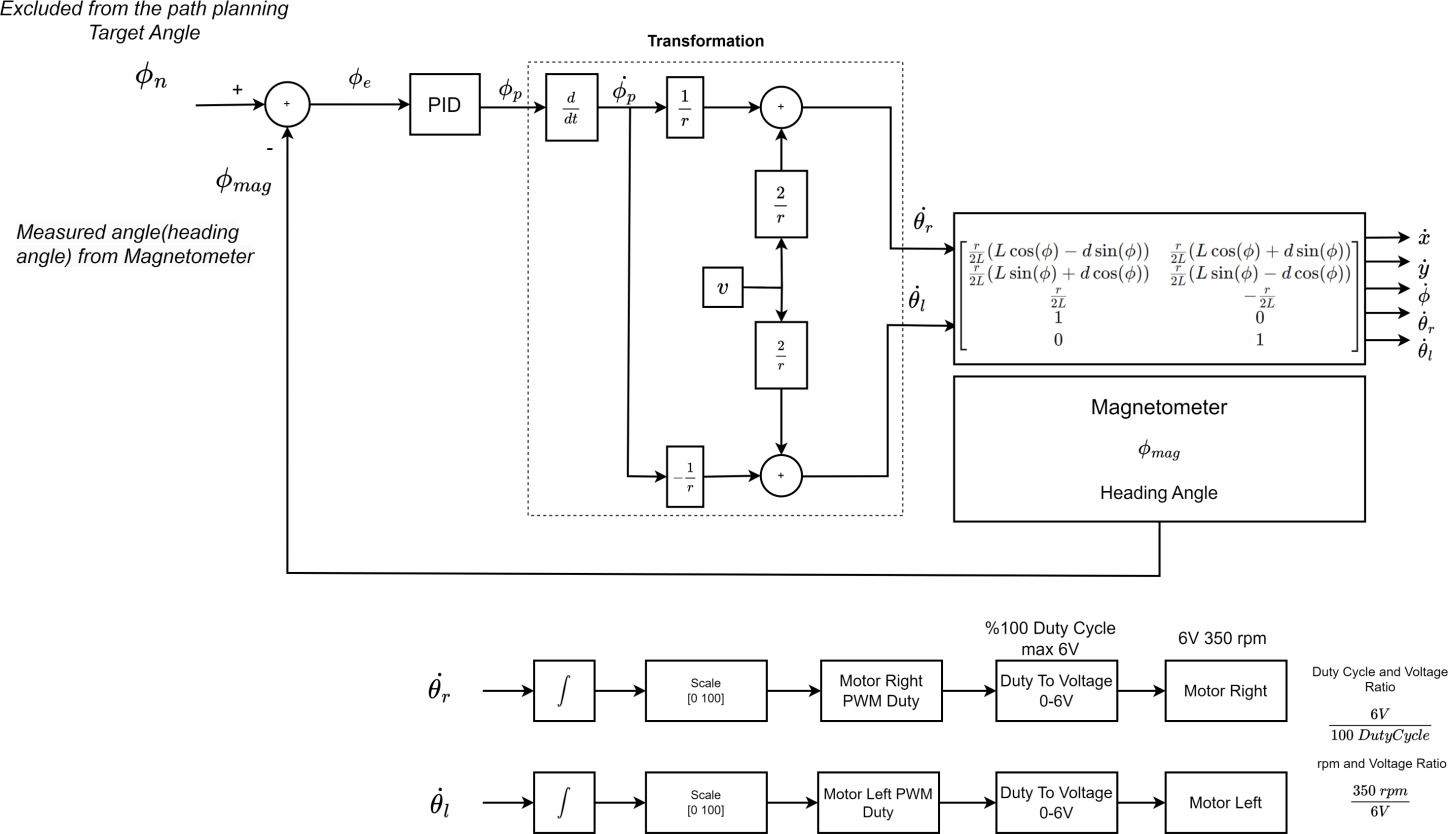
The control stage that will enable the AMR to heading towards the drivable area.

The PID controller is used to turn the mobile robot to the targeted angle. It sends a steering signal to the motor driver for the AMR to turn to the targeted angle. The structure of the controller is shown in Fig. 14. Here the set value is *θ*_*n*_. Here the set value is ∅_*n*_. The feedback value ∅_*mag*_ is the heading angle value of the mobile robot measured by the magnetometer. The head angle of the mobile robot measured by the magnetometer is the feedback value of the PID controller. The PID controller produces a PWM signal rate for AMR depending on the error value obtained according to the feedback value and the difference between the feedback value. This value takes a value between [0 100]. Duty Cycle ratio produces a speed of 350 rpm at max 6V to the right and left motor of the mobile robot. This speed is linearly adjusted according to the duty cycle and voltage ratio. Duty cycle and voltage ratio is $ \frac{6\mathrm{V}}{100\%} $. The speeds of the right and left wheels are also determined according to the ratio of the average voltage value. Speed and voltage ratio is $ \frac{350\mathrm{rpm}}{6\mathrm{V}} $. This value is converted to duty cycle value. PID coefficients were determined experimentally. According to the PWM ratios produced for the right and left wheels, the mobile robot is directed to the targeted angle.

### Experimental evaluation and comparisons

This section explains the results of the AMR drivable area estimation using the proposed method. In addition, comparative training and validation results of the Deeplabv3+ network according to different backbone architectures are examined. Metrics to evaluate the performance of the semantic segmentation network are explained. According to these metric results, the results for the high performance Deeplabv3+ network are examined for AMR’s of drivable area estimation. The proposed method has been implemented using the designed AMR.

### Metrics and experimental environment

In the proposed method, different standard metrics are used to measure the performance of the semantic segmentation network. These metrics are precision, recall, accuracy, F1-score and IoU (Intersection over Union) (Asgari Taghanaki et al., 2021). These metrics are explained in Eqs. (22)–(26).

(22) \begin{eqnarray*}recision& = \frac{TP}{TP+FP} \end{eqnarray*}

(23) \begin{eqnarray*}Recall& = \frac{TP}{TP+FN} \end{eqnarray*}

(24) \begin{eqnarray*}Accurucy& = \frac{TP+TN}{TP+TN+FP+FN} \end{eqnarray*}

(25) \begin{eqnarray*}F{1}_{Score}& =2. \frac{Precision.Recall}{Precision+Recall} \end{eqnarray*}

(26) \begin{eqnarray*}IoU& = \frac{TP}{TP+FP+FN} \end{eqnarray*}

where FN (false positive) is the number of true pixels that cannot be predicted. FP is the number of predicted pixels that fall outside the true masked pixels. TP is the number of intersections between the true positive true masked pixels and the predicted pixels. For true negative segmentation, TN is the number of correctly classified pixels. Metric measures are calculated based on the TP, TN, FP and FN numbers. The precision, recall, accuracy, F1-score and IoU metrics take values between 0 and 1. As each metric approaches 1, a better measure is obtained. These metrics are directly related. These metrics are often used to measure the performance of segmentation methods.

The Dice Loss function was used to train of the segmentation networks. It was used to measure the similarity between the two images. Dice Loss is expressed in Eq. (27), where *X* (Ground Truth) is the actual value label and $\hat {Y}$ is the predicted value label.

(27) \begin{eqnarray*}DiceLoss \left( y,\hat {p} \right) =1- \frac{2{|}X\cap Y{|}}{ \left\vert X \right\vert +{|}Y{|}} .\end{eqnarray*}

Intel Core i7-10700H processor @ 2.3 GHz Turbo, 16 GB DDR4 RAM and Nvidia RTX 3050 Ti 4GB GPU were used for training the segmentation models. The Pytorch and Scikit-Learn libraries in Python were used to implement the segmentation networks used in the proposed method. The trained segmentation is transferred learning to the specified AMR and the drivable area prediction is applied in real time on the Jetson Nano developer kit.

### Comparison of segmentation method and backbone architecture for mobile robot drivable path

The proposed method employs the DeeplabV3+ segmentation model, which is compared with UNet, FPN, PAN and PSPNet. Each segmentation model is trained with the same hyperparameters as DeeplabV3+, which were used in the proposed method. Furthermore, different backbone architectures that affect the success of the segmentation methods are compared. These include Resnet50, Resnet101, MobilenetV2, DPN and EfficientNet (Chen et al., 2017; Sandler et al., 2018; Tan & Le, 2019). The IoU, recall, precision, F1-score and accuracy metrics are employed to assess the efficacy of segmentation methods and backbone architectures. Furthermore, the dice loss results are employed to ascertain the losses incurred by the compared methods and backbone architectures. The results of this comparison indicate that the segmentation model and backbone architecture that yield the most optimal results should be applied in real time for AMR’s drivable path prediction. The metrics employed to assess the comparative outcomes are employed to ascertain the segmentation efficacy.

**Figure 15:**
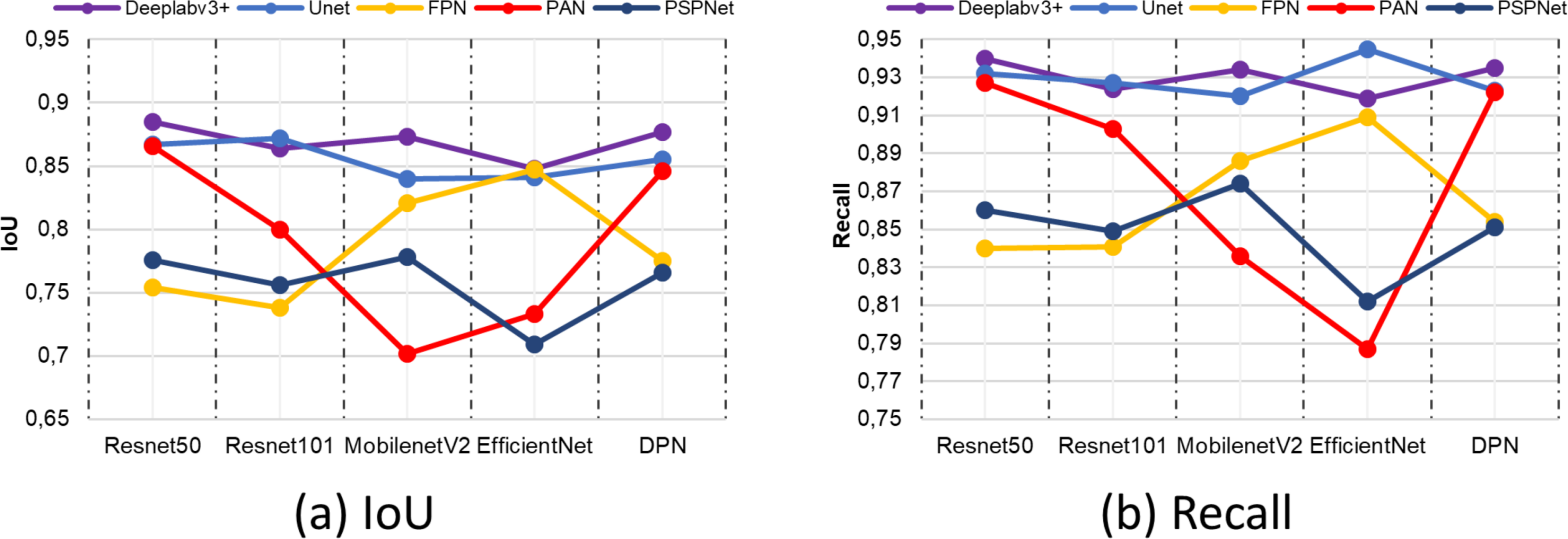
(A) IoU and (B) recall comparison results.

Figure 15 shows the IoU and Recall metric comparisons of different architectures and different backbone networks. IoU and Recall, which are among the metrics, are frequently preferred specially to evaluate semantic segmentation performance. According to the comparison results obtained, the Deeplabv3+ segmentation method achieved high performance by producing the closest value to 1 for each metric in all backbone networks. This value is valid for both IoU and recall metrics. The backbone network that provides the highest performance in the Deeplabv3+ method is resnet50.

**Figure 16:**
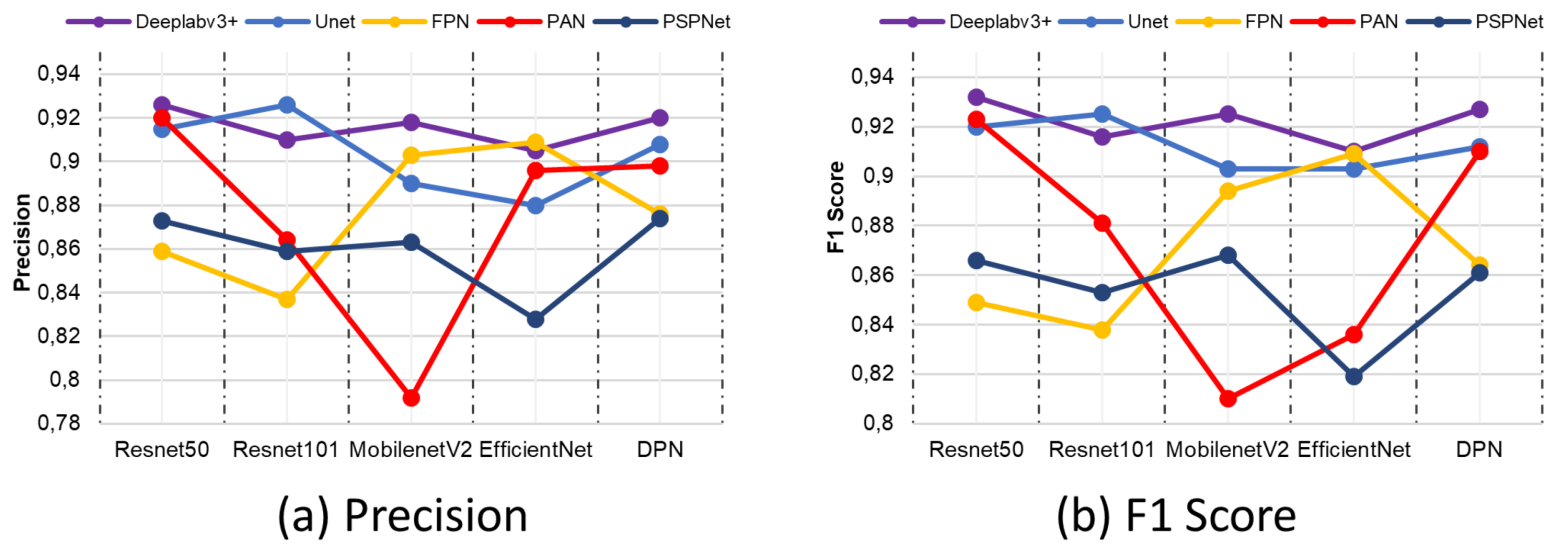
(A) Precision and (B) F1-score metric results.

In the prediction of correct pixels by semantic segmentation architectures and networks, the precision metric comes to the fore as a criterion for determining the correctly classified pixels in terms of the accuracy of the model. Similarly, the F1-score metric is a metric based on the relationship between recall and precision metric. When evaluated in terms of segmentation success, it is used in the evaluation of unbalanced data sets. Figure 16 shows the comparison results of precision and F1-score. According to the comparison results obtained, the combination of Deeplabv3 and Resnet50 shows the closest criteria to 1 in both the precision metric and the F1-score metric.

**Figure 17:**
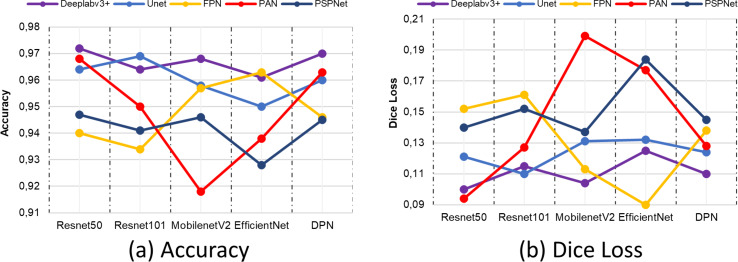
(A) Accuracy and (B) Dice Loss metric results.

One of the metrics employed in the general evaluation of semantic segmentation architectures in terms of correct classification is the accuracy metric. Although it carries a risk as a measure of an imbalanced distribution of classes, its evaluation in conjunction with other metrics taken in the study contributes to the reliability of the segmentation network. Figure 17A presents the comparative results of the accuracy metric. The results indicate that the Deeplabv3 and Resnet50 segmentation models exhibited the highest performance.

**Figure 18:**
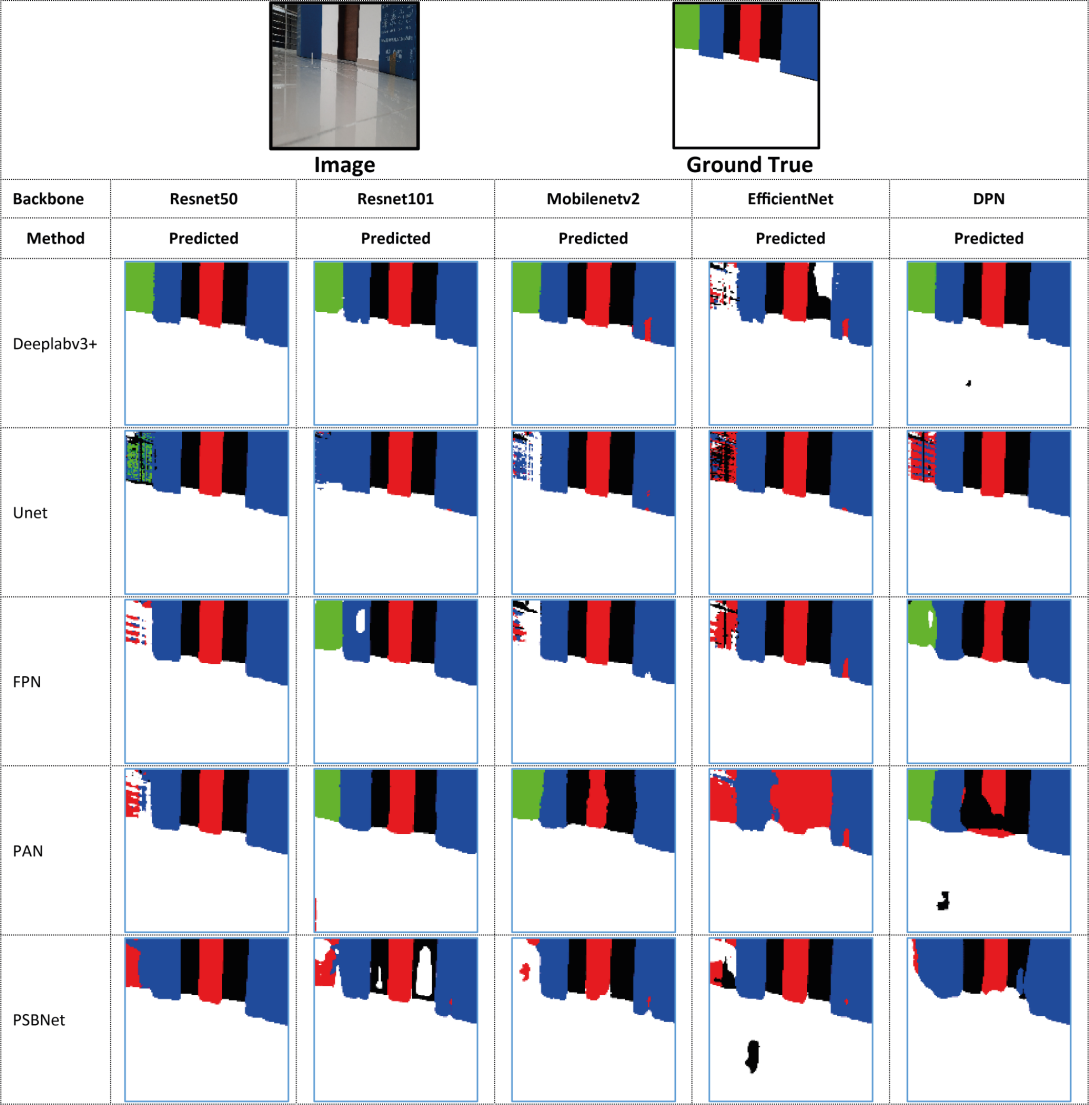
Comparison of predicted and drivable path marker results of the methods compared according to backbone networks.

In the comparison conducted on the same dataset, the losses of all segmentation models at the conclusion of the training process are also evaluated using the dice loss metric. Although the lowest loss values in the Deeplabv3+ model are obtained with the ResNet50 backbone network, the FPN+EfficienctNet and PAN+ResNet50 models have lower loss values. However, the Deeplabv3+ method is more successful in terms of segmentation performance according to the metrics obtained, despite this loss value. The combination of Deeplabv3+ and Resnet50 demonstrated the most optimal performance among the segmentation models and backbone architectures. Furthermore, the results of a sample position image taken from the prediction results of the methods compared according to the backbone networks are presented in Fig. 18. The results obtained are presented in the form of ground truth, predicted results, and drivable area according to each method compared. As illustrated in Fig. 18, the proposed Deeplabv3+ method exhibits high accuracy and performance.

### Comparison of real-time accuracy and FPS in terms of training model performance

In order to evaluate the real-time performance of the training model and the segmentation accuracy of the model, they are compared in terms of FPS. According to the Deeplabv3+ and Resnet50 backbone network used in the proposed model, other models are considered according to the Resnet50 backbone network. Table 3. The comparison of the model in terms of performance in terms of real-time accuracy and FPS is expressed.

**Table 3:** Performance comparison of trained models.

**Model + Backbone**	**mIoU**	**FPS**
Deeplabv3+ / Resnet50	0.885	8.81
Unet / Resnet50	0.867	8.26
FPN / Resnet50	0.754	8.58
PAN / Resnet50	0.866	8.35
PSBNet/ Resnet50	0.776	23.54

The accuracy of the trained segmentation model is an important criterion parameter for mIoU. Although accuracy is an important criterion, the processing load for real-time applications resulting from the number of parameters of the trained model is also critical in terms of functionality. Therefore, it is expected that these two parameters are balanced. The Deeplabv3+ model shows the best performance in terms of accuracy. However, although this model does not show the best value in terms of FPS, it has produced similar results to other trained models. The number of image frames of the proposed method and the FPS values given between the PID controller unit are directly related. When evaluated in this respect, the PID controller is directly executed by the central processing unit run by Jetson Nano and the segmentation result generation time of the trained model is much longer than this sample time.

### Implementation and validation

**Figure 19:**
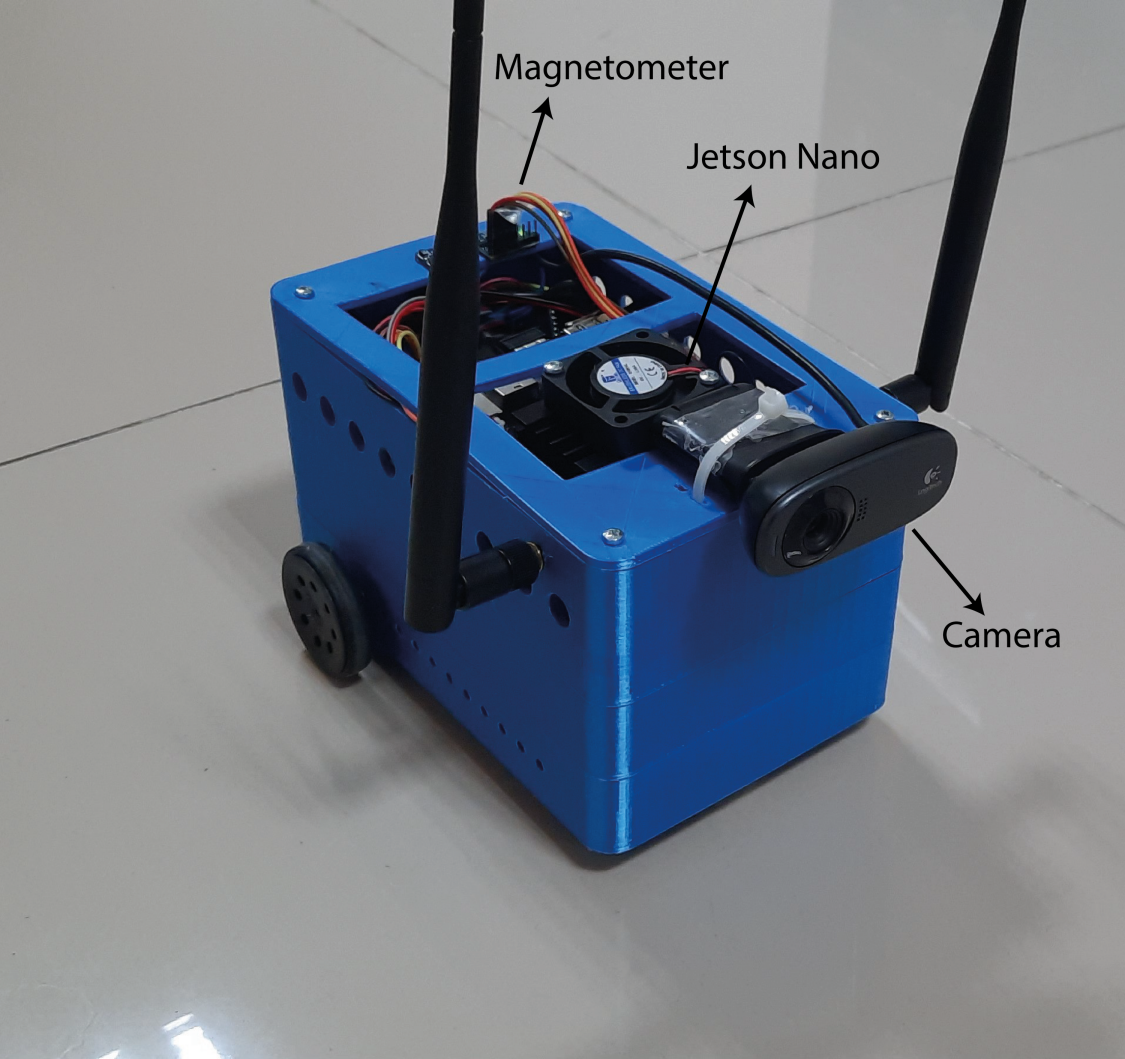
AMR.

To validate the proposed Deeplabv3+ based method, an application is made in a closed indoor environment in a real-world environment. The implemented experiments are carried out with the AMR whose design and specifications are described. The AMR equipped with Jetson nano is shown in Fig. 19.

**Figure 20:**
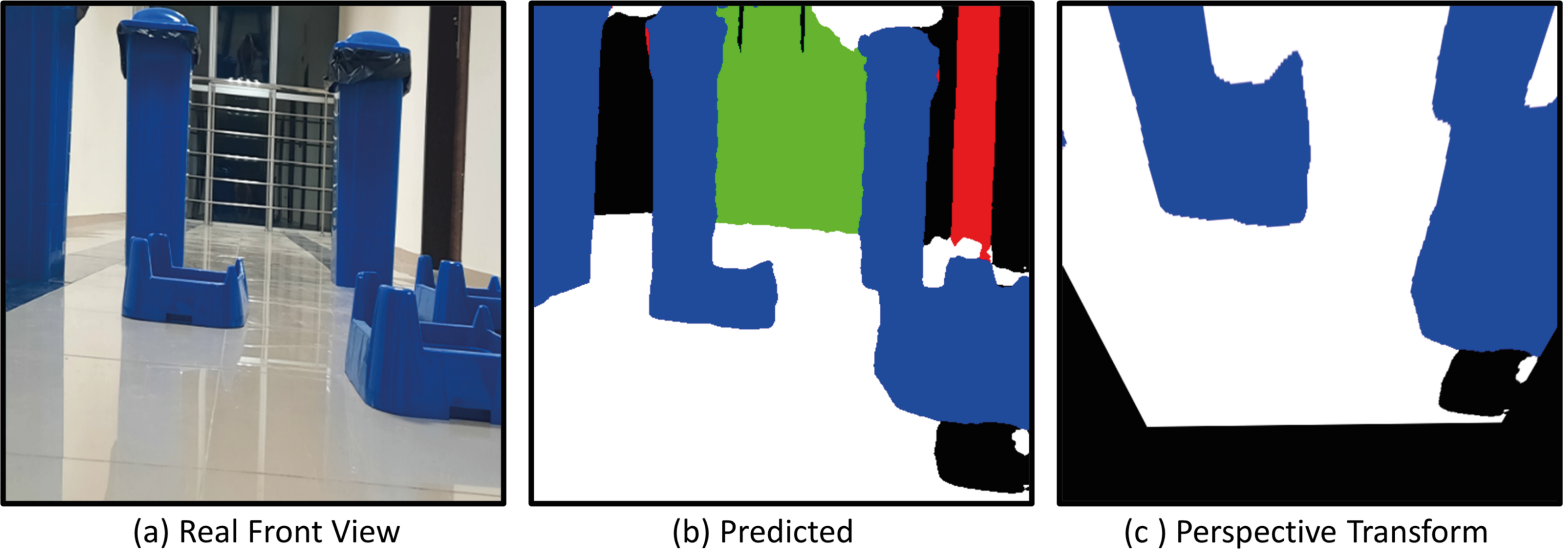
(A) Real front view, (B) predict and (C) perspective Transform process of the mobile robot in a challenging scenario with obstacles for Scenario-1.

The model with the proposed Deeplabv3+ based Resnet50 backbone network is implemented on AMR. In the indoor environment, complexly placed boxes and doors that limit the environment are obstacles. In the applications, the Deeplabv3+ based method is used to predict the drivable path of the mobile robot. For this, the drivable path detection results of the AMR from several different positions in the specified indoor environment are examined. In the indoor environment, there are doors, garbage boxes, walls, windows and drivable areas in the labels and colors on which the mobile robot was trained. The application was carried out in an indoor environment with different light levels (such as shadowy areas). The masked images obtained from the proposed Deeplabv3+ network and resnet50 backbone architecture play a significant role in directing the AMR to the drivable area. In order to verify the proposed method, a challenging scenario including the transformation from the image perceived by the mobile robot from any point in a difficult environment full of obstacles to segmentation and perspective transformation is shown in Fig. 20. This process allows the mobile robot to make sense of the environment it is in through semantic segmentation. After the perspective transformation, the mobile robot becomes able to produce a navigation strategy. Afterwards, the mobile robot can plan a path to the desired final location according to the perspective transformed image.

**Figure 21:**
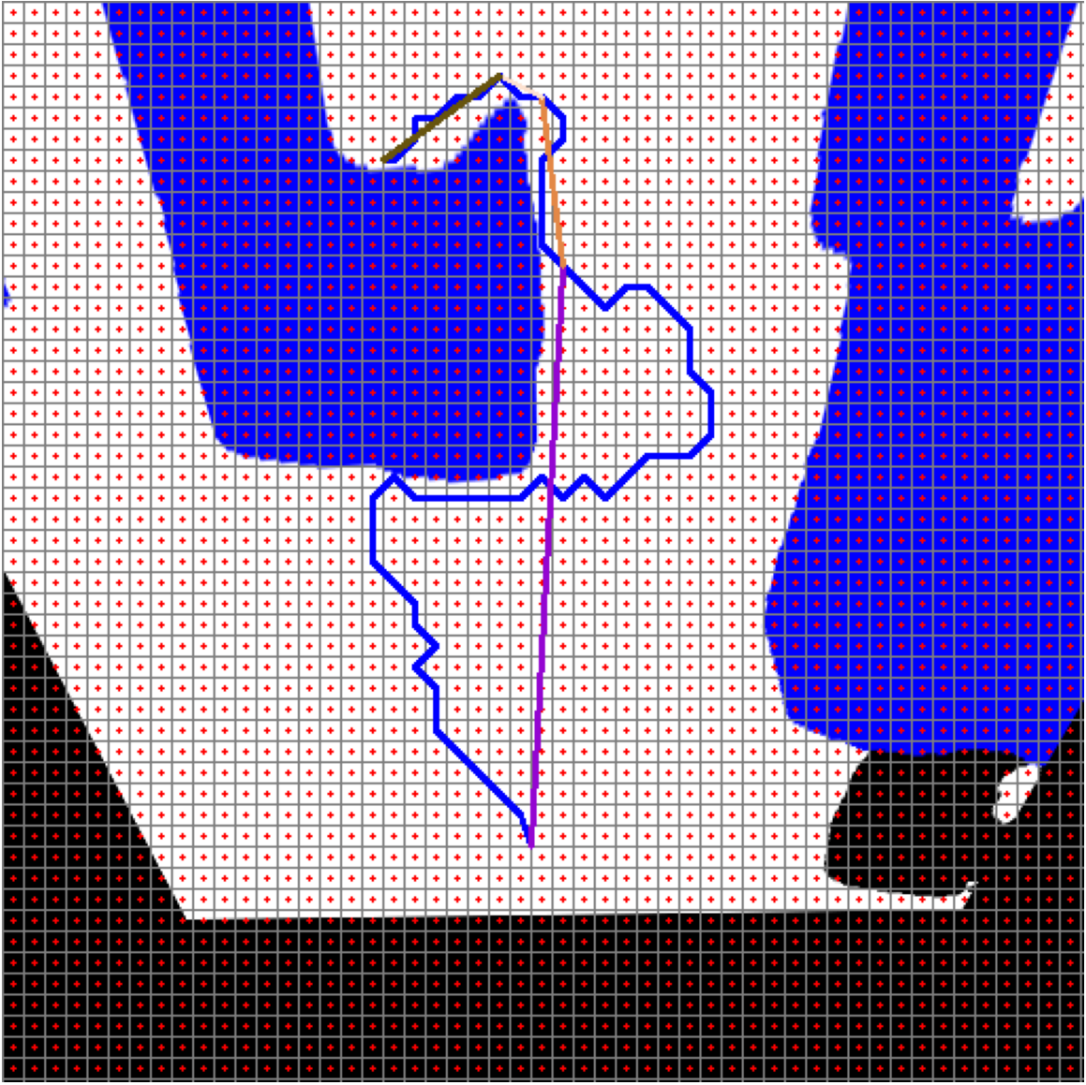
RRT*(blue) and smooth path planning for Scenario-1.

Figure 21 shows the path planning process of the mobile robot according to Scenario-1. The mobile robot generates the path plan with grid-based RRT* according to the image it perceives at its current location. The path generated with RRT* is smoothed. The smooth process also makes it easier to plan the proportional movement of the mobile robot with the ground.

**Figure 22:**
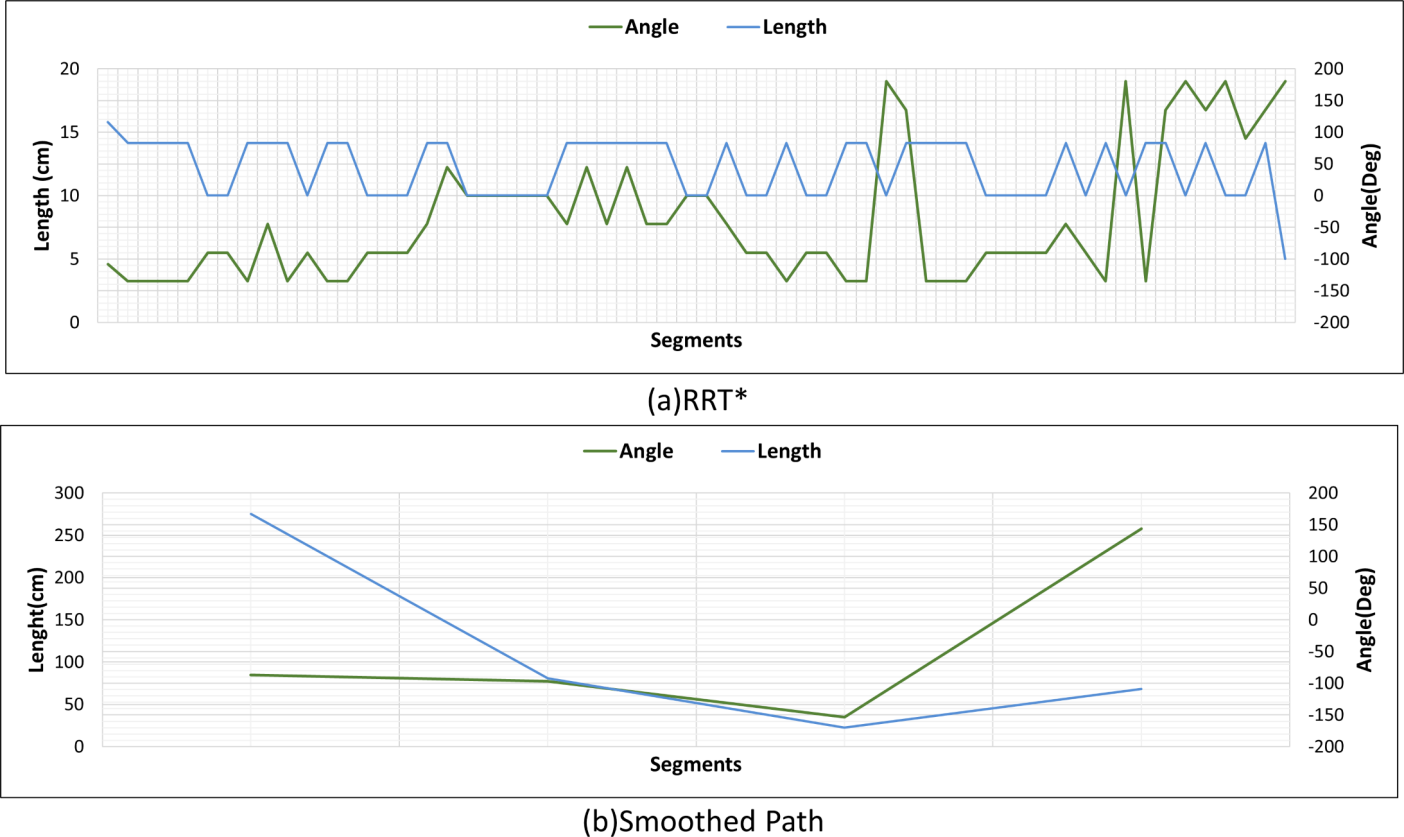
RRT* and smoothed path angle and length changes for Scenario-1.

Figures 22A and 22B show the angle produced by both RRT* and smoothed path on the path followed by the robot in scenario-1 according to the proposed method and the angle of the transition of segments between each grid. The path planning made with RRT* has a path transition between grids consisting of 60 segments. The mobile robot is exposed to different angle changes in each segment transition. In addition, the path lengths of the segments are quite short.

Figure 22B shows the angle and length changes according to the segments created for the smoothed version of the path plan determined by RRT*. The smoothed path also consists of 4 segments. The lengths between the segments are longer than those in RRT*. In addition, when the angle changes are compared, the angle changes of the smoothed path plan are less than those in RRT*.

**Figure 23:**
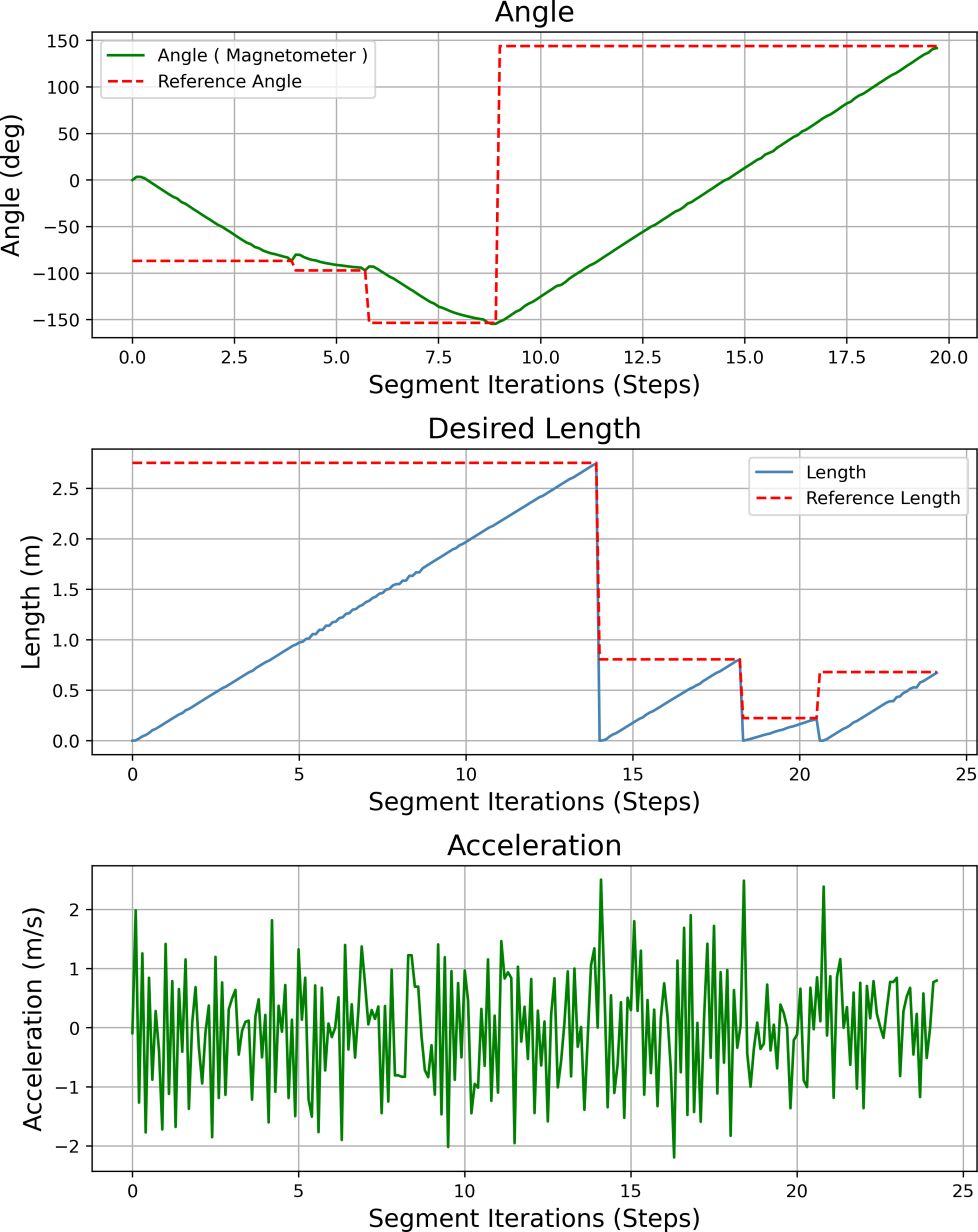
Angle, length, magnetometer and acceleration measurement results of the mobile robot for the path planning according to Scenario-1.

Figure 23 shows the heading angle, target angle, target distances and acceleration results of the path planning of the mobile robot in Scenario-1. The results obtained from different segments due to path planning are expressed separately. Desired length starts from 0 (zero) after each segment. Acceleration values reach the maximum value at the beginning of each segment.

**Figure 24:**
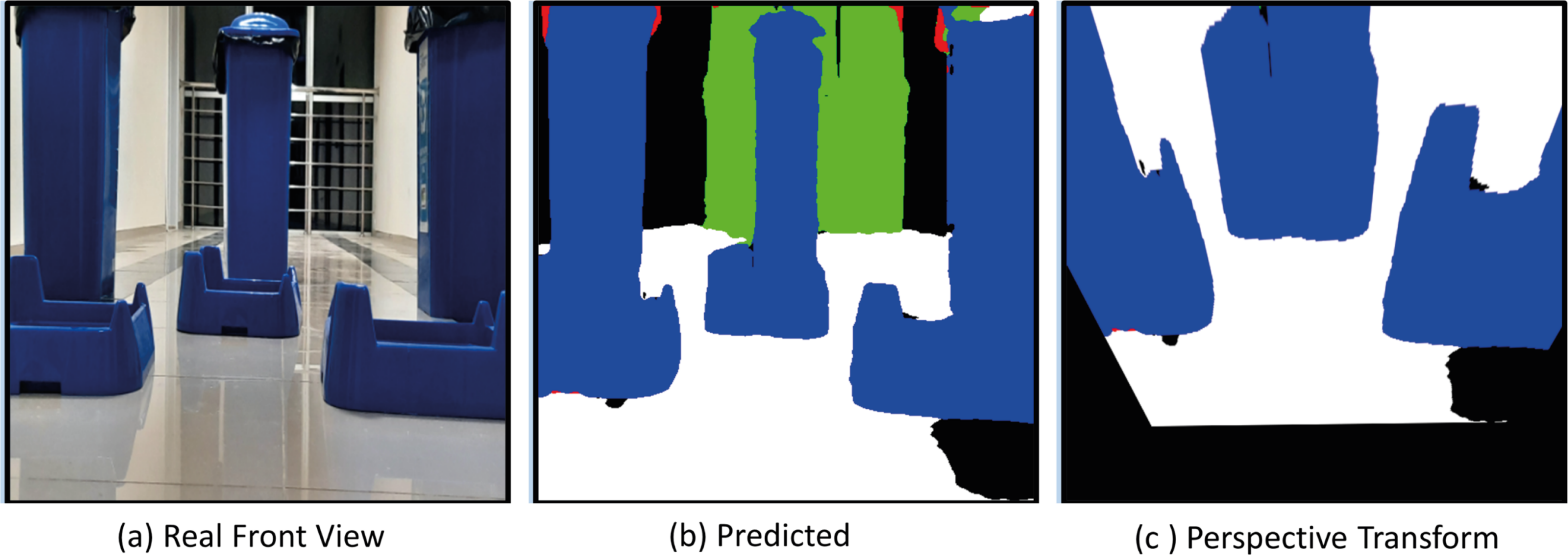
(A) Real front view, (B) predict and (C) perspective Transform process of the mobile robot in a challenging scenario with obstacles for Scenario-2.

Figure 24 shows the drivable area detection and path planning process for scenario-2. In this challenging scenario, the path planning process obtained by the mobile robot in a different position is discussed. What is striking here is the segmentation success of Deeplabv3+. The accuracy of the segmentation of the mobile robot affects both path planning and safe driving.

**Figure 25:**
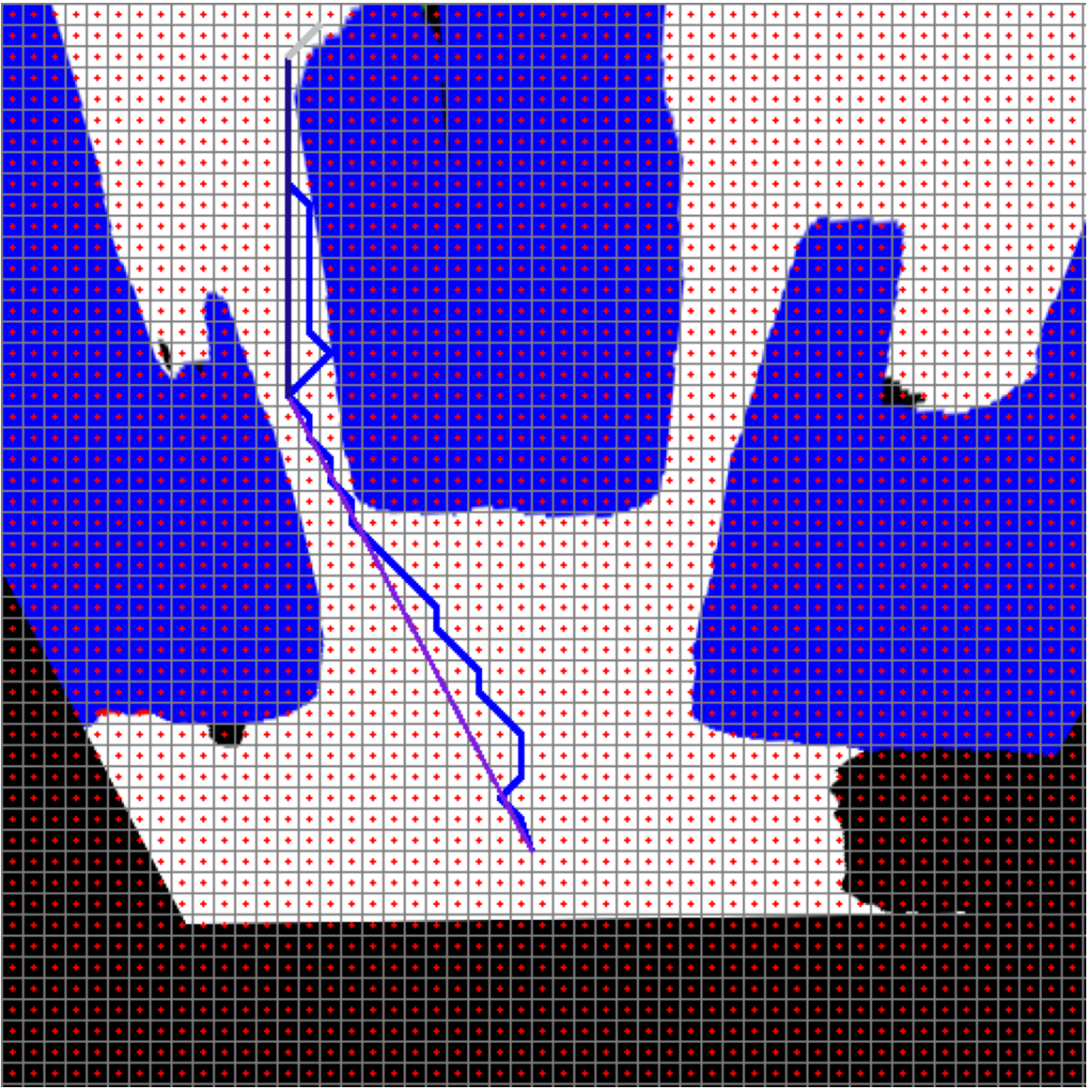
RRT*(blue) and smooth path planning for Scenario-2.

In Scenario-2, the mobile robot can plan a path that can pass through narrow spaces after the perspective transform. Figure 25 shows the RRT* and smoothed path plans for Scenario-2.

**Figure 26:**
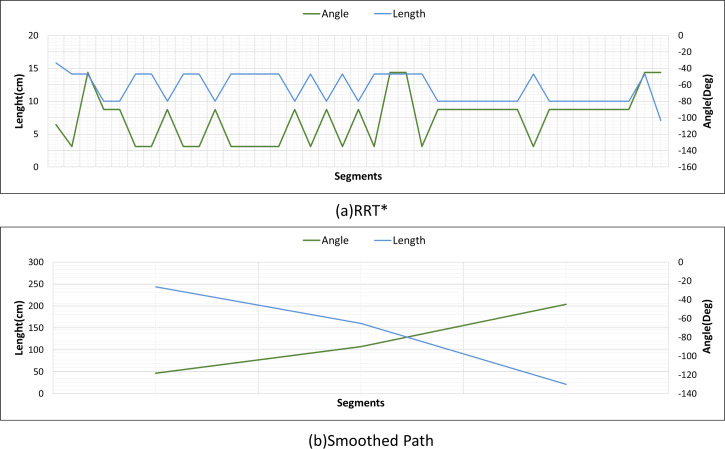
RRT* and smoothed path angle and length changes for Scenario-2.

Figure 26 shows the comparison of RRT* and smoothed path for the path plan obtained in Scenario-2. The path created with RRT* is created from 39 grid segments, while the smoothed path consists of three segments. The smoothed path enables the path extracted with RRT* to reach the targeted position with fewer turns.

**Figure 27:**
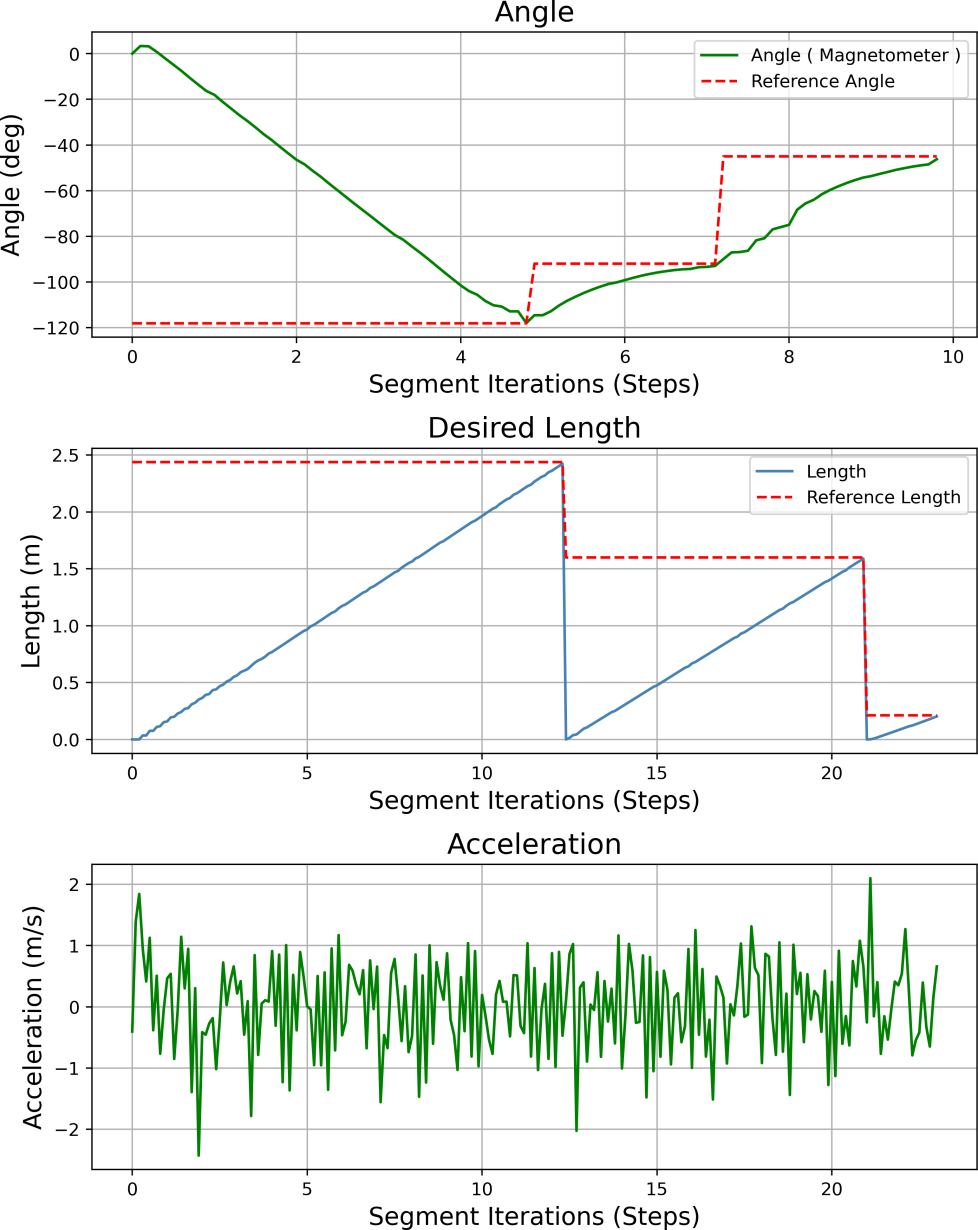
Angle, length, magnetometer and acceleration measurement results of the mobile robot for the path planning according to Scenario-2.

Figure 27 shows the heading angle, target angle, target distances and acceleration results of the mobile robot’s path planning according to Scenario-2. In three different segments, the mobile robot reaches the target.

**Figure 28:**
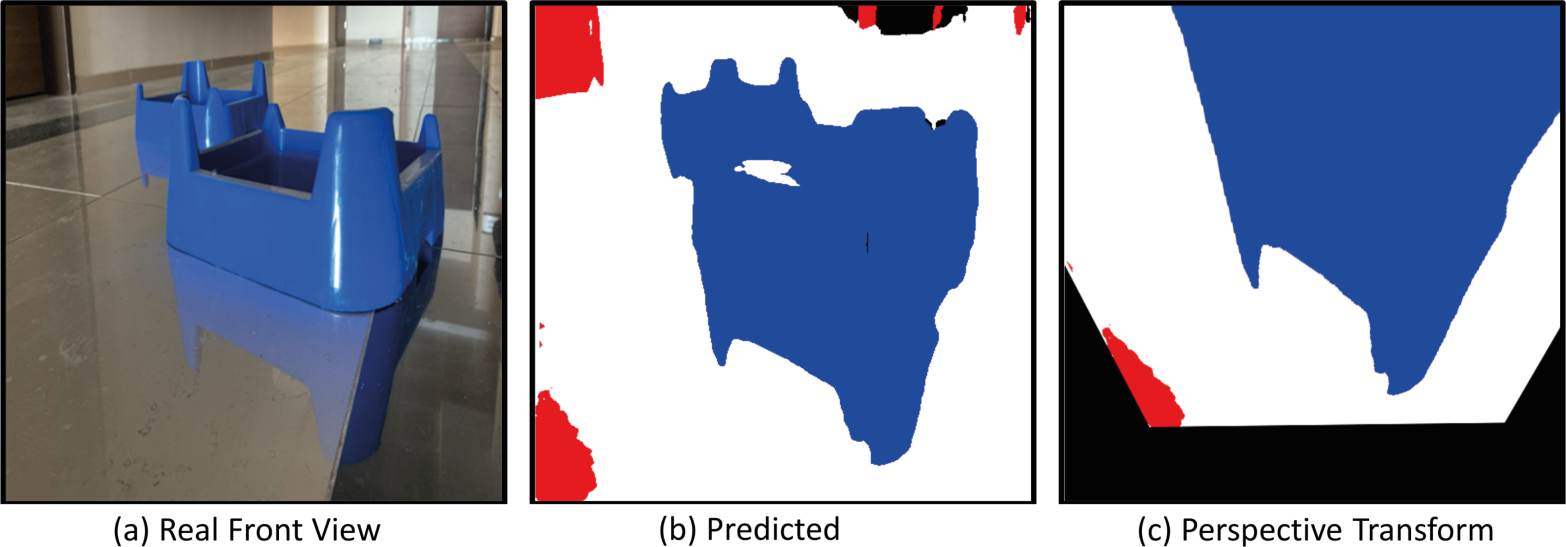
(A) Real front view, (B) predict and (C) perspective Transform process of the mobile robot in a challenging scenario with obstacles for Scenario-3.

Figure 28 shows the real front view, Predicted and perspective transform process of the mobile robot for Scenario-3. In Scenario-3, the path planning and drivable path determination of the mobile robot in a position relatively closer to the obstacles are considered.

**Figure 29:**
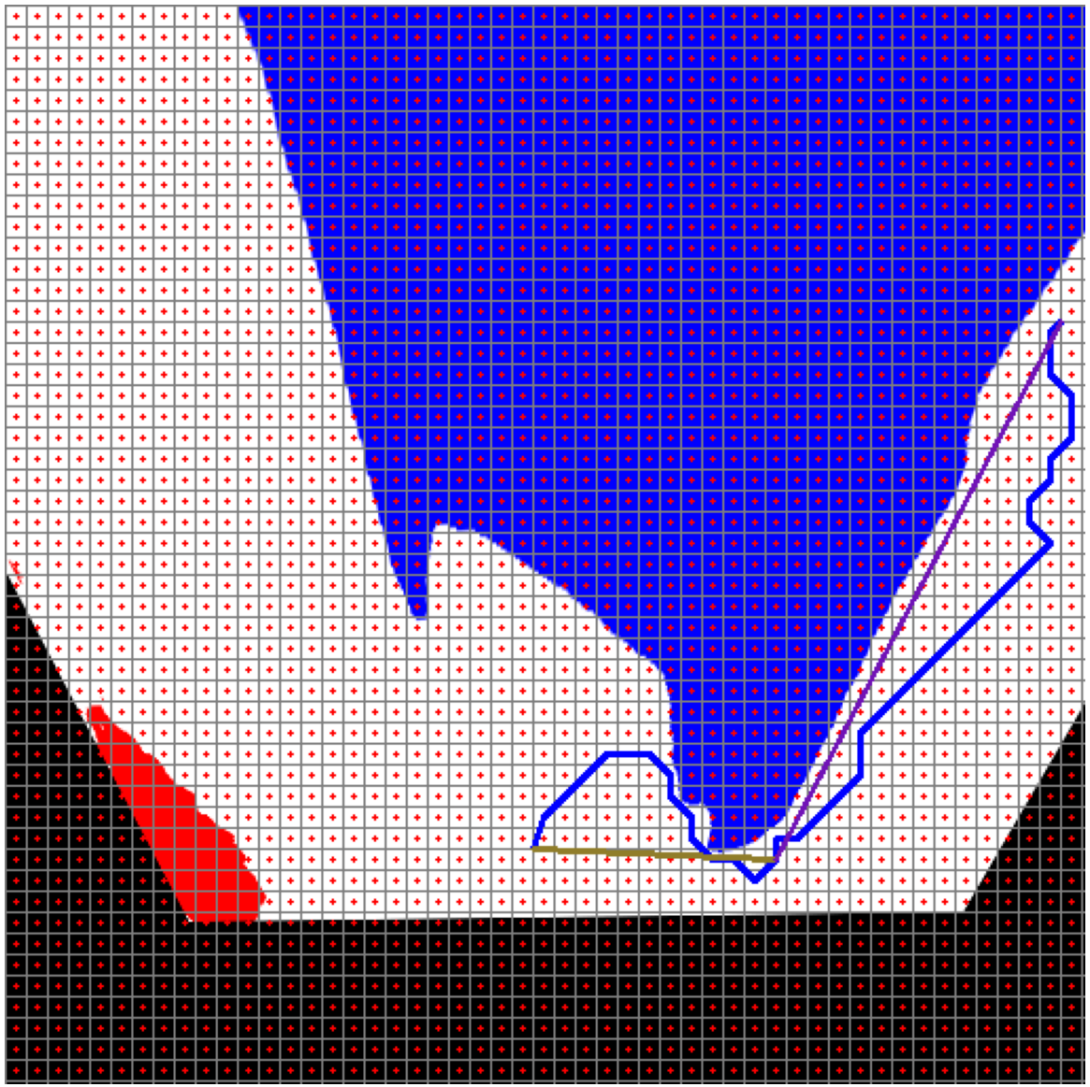
RRT*(blue) and smooth path planning for Scenario-3.

In Scenario-3, when the mobile robot is close to the obstacle, it plans the path based on the grid-based RRT* perspective transformation to orient the robot to the specified location. Figure 29 shows the RRT* and smoothed path for the near obstacle scenario.

**Figure 30:**
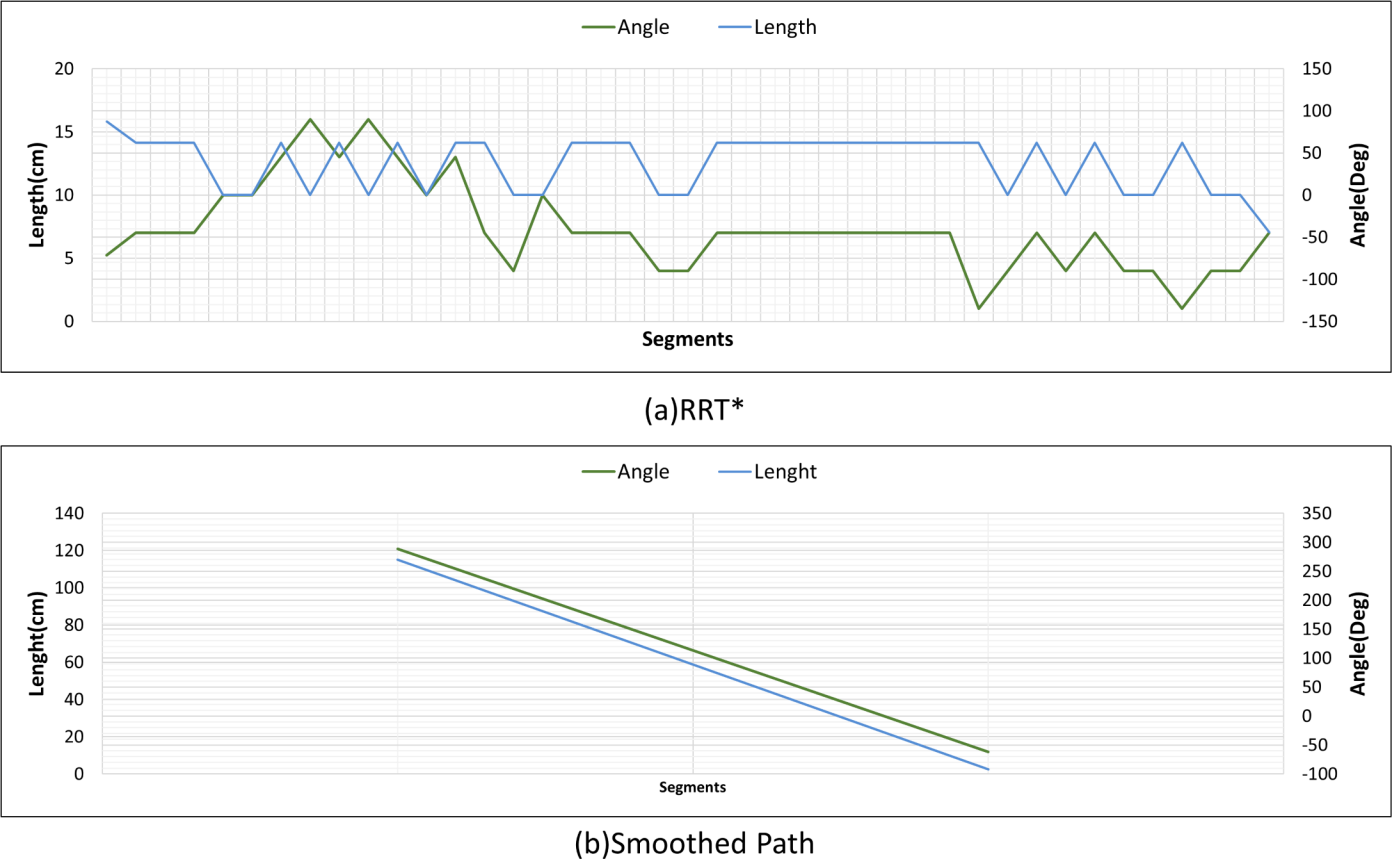
RRT* and smoothed path angle and length changes for Scenario-3.

Figure 30 shows the angle and length changes of the mobile robot for Scenario-3. Although the mobile robot is close to the obstacle, the smoothed path creates very few turning angles according to RRT*.

**Figure 31:**
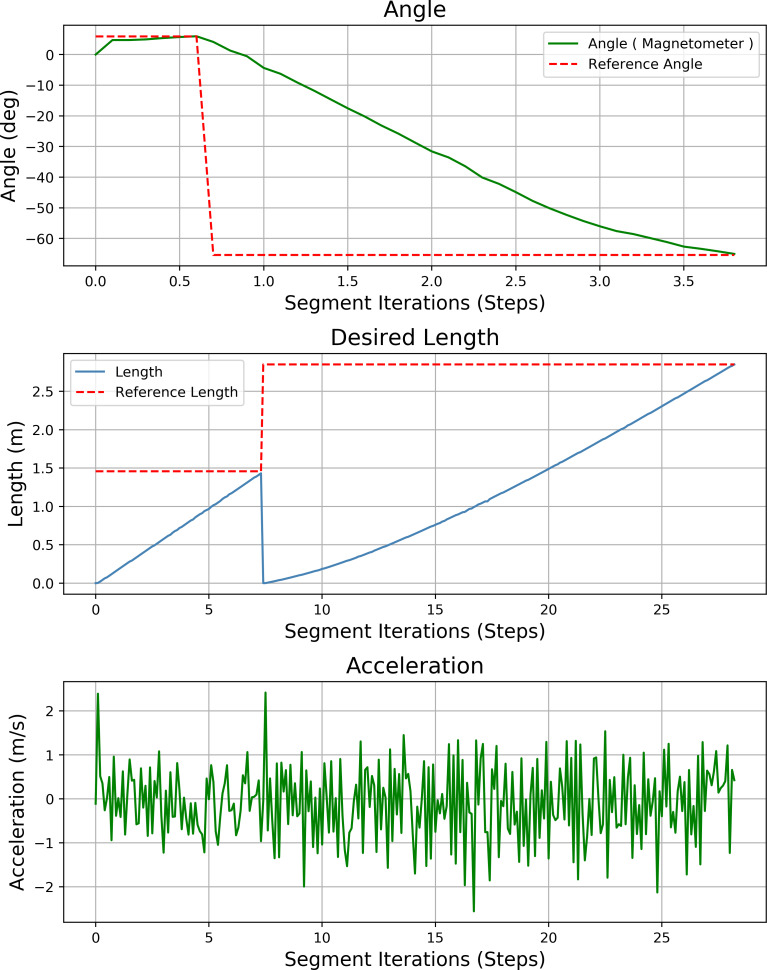
Angle, length, magnetometer and acceleration measurement results of the mobile robot for the path planning according to Scenario-3.

Figure 31 shows the heading angle, target angle, target distances and acceleration results of the mobile robot’s path planning according to Scenario-3. In two different segments, the mobile robot is directed to the target.

 In the presented scenario, the AMR creates an angle to avoid the obstacles it encounters and steers towards this angle. In addition to steering towards the drivable area, the mobile robot avoids limited turning points, turning angles with high risk of hitting obstacles, and produces steering towards the most suitable path to move. In the application made with the designed AMR, results are given from environment images at different light levels. This is also an indicator of the accuracy of the mobile robot in real world conditions. The developed drivable path determination method enables the determination of the appropriate path that will support the autonomous navigation of the mobile robot.

## Conclusion

In this study, a navigation strategy using the grid-based RRT* algorithm was developed using the Deeplav3+ segmentation model to determine the drivable path of the AMR. The method provides the determination of a safe and collision-reducing drivable path to help the navigation of the mobile robot. Gaussian filter was used to increase the segmentation success and multi-otsu thresholding was used to improve the multi-class segmentation. After the drivable path was determined, the scene perceived by the mobile robot was transformed in the real world space using the perspective transform so that the mobile robot could plan its path to the targeted location. Then, the images were divided into grids to be used in the path planning of the mobile robot. The path planning to the targeted location was done with the grid-based RRT* algorithm.

Deeplav3+ segmentation network used in determining the arable area and Resnet50, Resnet101, MobileNetv2, EfficientNet and DPN backbone architectures were compared with Unet, FPN, PAN and PSPnet segmentation network models. According to the recall metric, it performed 1.70% better than Resnet101, 0.64% better than Mobilenetv2, 2.23% better than EfficientNet and 0.53% better than DPN. IoU, recall, precision, F1-score, accuracy and Dice Loss performance metrics were used to evaluate the comparison results. The proposed Deeplabv3+ and Resnet50 architecture outperformed Resnet101 by 2.37%, Mobilenetv2 by 1.36%, EfficientNet by 4.18% and DPN by 0.9% according to IoU metric. It performed 1.73% higher than the precision metric, 0.86% higher than Mobilenetv2, 2.27% higher than EfficientNet and 0.65% higher than DPN. It performed 1.72% higher than F1-score metric, 0.75% higher than Mobilenetv2, 2.36% higher than EfficientNet and 0.54% higher than DPN. It performed 0.82% higher than accuracy metric, 0.41% higher than Mobilenetv2, 1.13% higher than EfficientNet and 0.21% higher than DPN.

To validate the proposed method, a two-wheeled mobile robot equipped with jetson nano is designed. A path following strategy using acceleration and magnetometer sensors is used to implement the proposed navigation strategy. In order to test the developed method, three different challenging scenarios were implemented. RRT* and smoothed path planning results are analyzed.

The limitations that may occur in this study are as follows: The classes that make up the dataset are limited to drivable paths, windows, walls, doors and bin boxes. This shows that the possibility of different objects in the environment where the mobile robot moves is ignored. This situation can lead to unclassified objects being confused with the defined classes. In addition, the developed method ignores the movement of dynamic objects. For static objects, the objects are segmented. Since the dataset of captured images is made in a corridor environment, the mobile robot’s drivable path detection is valid for this environment.

Future work aims to develop a method that can perform segmentation with high accuracy in an environment with dynamic obstacles. In addition, an improved CNN network architecture will be designed to increase the accuracy of the semantic segmentation network used in the drivable path detection method.

## Additional Information and Declarations

### Data Availability

The following information was supplied regarding data availability: The data is available at figshare: misir, Oguz (2024). Drivible-Area-Semantic-Segmentation-Raw-Data-for-Mobile-Robot. figshare. Figure. https://doi.org/10.6084/m9.figshare.27061246.v2.
